# The FZD7‐TWIST1 axis is responsible for anoikis resistance and tumorigenesis in ovarian carcinoma

**DOI:** 10.1002/1878-0261.12425

**Published:** 2019-01-19

**Authors:** Ming Tan, Mohammad Asad, Valerie Heong, Meng Kang Wong, Tuan Zea Tan, Jieru Ye, Kuee Theng Kuay, Jean Paul Thiery, Clare Scott, Ruby Yun‐Ju Huang

**Affiliations:** ^1^ Cancer Science Institute of Singapore Singapore; ^2^ Center for Translational Medicine National University of Singapore Singapore; ^3^ Department of Obstetrics and Gynaecology National University Hospital of Singapore Singapore; ^4^ Department of Haematology‐Oncology National University Cancer Institute Singapore Singapore; ^5^ Walter and Eliza Hall Institute of Medical Research Parkville Australia; ^6^ Department of Biochemistry National University of Singapore Singapore; ^7^ Institute of Molecular and Cell Biology A*STAR Singapore Singapore; ^8^ Department of Anatomy Yong Loo Lin School of Medicine National University of Singapore Singapore

**Keywords:** anoikis, BCL2, FZD7, ovarian carcinoma, tumorigenesis, TWIST1

## Abstract

Frizzled family receptor 7 (*FZD7*), a Wnt signaling receptor, is associated with the maintenance of stem cell properties and cancer progression. FZD7 has emerged as a potential therapeutic target because it is capable of transducing both canonical and noncanonical Wnt signals. In this study, we investigated the regulatory pathway downstream of *FZD7* and its functional roles. We found that *FZD7* expression was crucial to the maintenance of the mesenchymal phenotype, anoikis resistance, and spheroid and tumor formation in ovarian cancer (OC). We identified *TWIST1* as the crucial downstream effector of the *FZD7* pathway. *TWIST1*, a basic helix loop helix transcription factor, is known to associate with mesenchymal and cancer stem cell phenotypes. Manipulating *TWIST1* expression mimicked the functional consequences observed in the *FZD7* model, and overexpression of *TWIST1* partially rescued the functional phenotypes abolished by *FZD7* knockdown. We further proved that *FZD7* regulated *TWIST1* expression through epigenetic modifications of H3K4me3 and H3K27ac at the *TWIST1* proximal promoter. We also identified that the *FZD7*‐*TWIST1* axis regulates the expression of *BCL2*, a gene that controls apoptosis. Identification of this *FZD7*‐*TWIST1*‐*BCL2* pathway reaffirms the mechanism of anoikis resistance in OC. We subsequently showed that the *FZD7*‐*TWIST1* axis can be targeted by using a small molecule inhibitor of porcupine, an enzyme essential for secretion and functional activation of Wnts. In conclusion, our results identified that the *FZD7*‐*TWIST1* axis is important for tumorigenesis and anoikis resistance, and therapeutic inhibition results in cell death in OCs.

**Abbreviations**
CAM: chorioallantoic membrane
CSC: cancer stem cells
ECM: extracellular membrane
ED: embryonic day
EMT: epithelial to mesenchymal transition
EOC: epithelial ovarian cancer
FACS: florescence‐activated cell sorting
FZD7: frizzled 7
FZD: frizzled
IgG: immunoglobulin G
Mes: mesenchymal
OC: ovarian cancer
PCP: planar cell polarity
qPCR: quantitative PCR
shFZD7: short hairpin against FZD7
shLuci: short hairpin luciferase
siRNA: small interfering RNA
TSS: transition start site

## 1 Introduction

The Wnt signaling pathway comprises of Wnt ligands that are secreted glycoprotein and frizzled (FZD) receptors, which are seven‐pass transmembrane receptors with an extracellular N‐terminal cysteine‐rich domain (Bhanot *et al*., 1996). Wnt signaling involves two pathways, namely the canonical and noncanonical pathway (Veeman *et al*., 2003). In the canonical Wnt pathway, the T‐cell factor/lymphoid enhancer factor‐1 and β‐catenin act as a core regulator through nuclear translocation and transcriptional activation of target genes critical for cancer cell stemness and differentiation (Reya and Clevers, 2005; Reya *et al*., 2003; Yu *et al*., 2013). The noncanonical Wnt pathway can be further divided into two distinct pathways, Wnt/planar cell polarity (PCP) and Wnt/calcium (Ca^2+^) pathway. In the Wnt/PCP pathway, binding of Wnt ligands to FZD receptors stimulates the activities of the small GTPases Rho, Rac, and cdc42, which regulates diverse processes, such as cell polarity and migration (Nobes and Hall, 1999; Raftopoulou and Hall, 2004). An aberration in Wnt/PCP pathway has been shown to enhance cell proliferation, survival, abnormal tissue polarity, and cell invasion and cancer metastasis (Katoh, 2005; Ridley, 2004). In the Wnt/Ca^2+^ pathway, an increase in the level of intracellular Ca^2+^ is triggered by Wnt‐FZD binding and activation of phospholipase C via G protein. The increase of intracellular Ca^2+^ also activates protein kinase C. Deregulation of the Wnt/Ca^2+^ pathway has been implicated to drive cellular motility, cellular proliferation, and epithelial–mesenchymal transition (EMT) during cancer progression (Dissanayake *et al*., 2007; Wang *et al*., 2010) .

Frizzled family receptor 7 (*FZD7*) is one of the *FZD* receptors that mediates both canonical and noncanonical Wnt signaling (Abu‐Elmagd *et al*., 2006; Zhang *et al*., 2013). It is an important regulator of pluripotency resulting in the undifferentiated phenotype (Fernandez *et al*., 2014; Melchior *et al*., 2008) in embryonic stem cells and also regulates cellular proliferation in human embryonal carcinoma cells. *FZD7* contributes to cell stemness in several normal and cancer cells (Chakrabarti *et al*., 2014; Mei *et al*., 2014; Song *et al*., 2006). Aberrant activation of *FZD7* has been found in several types of cancer such as breast (Yang *et al*., 2011), colorectal (Ueno *et al*., 2009), hepatocellular carcinoma (Merle *et al*., 2005), and gastric cancer (Kirikoshi *et al*., 2001). Recently, it has been shown that *FZD7* regulates spheroid proliferation in ovarian cancer stem cells (CSCs) (Condello *et al*., 2018). Our group previously showed that *FZD7* drives *in vitro* aggressiveness in ovarian cancer (OC) via the noncanonical Wnt/PCP pathway (Asad *et al*., 2014) while inhibition of FZD receptors through antibody binding reduced tumor‐initiating cell populations in a range of tumor types (Gurney *et al*., 2012) making the FZD7 pathway very intriguing and warrants further exploration of its regulatory mechanisms and potential for therapeutic targeting.

*TWIST1*, a transcription factor with a basic helix loop helix domain (Thisse *et al*., 1988), plays important roles in EMT and cancer metastasis. *TWIST1* recruits the nucleosome remodeling and deacetylase complex to upregulate mesenchymal (Mes) markers, to repress epithelial genes, and therefore to induce EMT (Qin *et al*., 2012). It has been shown to cause apoptosis resistance (Ansieau *et al*., 2008; Maestro *et al*., 1999; Valsesia‐Wittmann *et al*., 2004), chemoresistance (Cheng *et al*., 2007; Li *et al*., 2009), enrichment of CSC populations (Battula *et al*., 2010; Mani *et al*., 2008; Vesuna *et al*., 2009), and cell invasiveness (Yang *et al*., 2004). Several groups have reported the association of *TWIST1* with increased tumorigenicity in breast (Yang *et al*., 2004), prostate (Yuen *et al*., 2007), and gastric cancers (Feng *et al*., 2009; Luo *et al*., 2008). In OC, *TWIST1* overexpression correlated with poorer clinical outcomes (Hosono *et al*., 2007). It has also been linked to both the canonical and noncanonical Wnt pathways. In the canonical pathway, *TWIST1* acts as a downstream effector of Wnt3a (Reinhold *et al*., 2006), Wnt1 (Howe *et al*., 2003), and β‐catenin (Goodnough *et al*., 2016), while in the noncanonical pathway, high expression of *TWIST1* correlates with the expression of FZD receptor 6 (*FZD6*), which is associated with increased spheroid‐forming activity and poor survival in neuroblastoma (Cantilena *et al*., 2011).

Ovarian carcinoma is a highly heterogeneous entity with multiple genetic aberrations and distinct molecular subtypes (Cancer Genome Atlas Research Network, 2011; Tan *et al*., 2013; Tothill *et al*., 2008). During trans‐coelomic metastasis of OC, detachment of OC cells from the primary site via an EMT‐like mechanism (Ahmed *et al*., 2010; Chua *et al*., 2011; Davidson *et al*., 2012) allows the dissemination to occur. EMT describes a transition from polarized epithelial cells to motile and invasive Mes cells (Nieto *et al*., 2016), while anoikis refers to apoptosis induced by lack of proper cell/extracellular membrane (ECM) attachment (Gilmore, 2005). As OC cells tend to shed from primary tumors and suspend in ascites to form secondary lesions throughout the peritoneal cavity, anoikis resistance is considered as one of the most important features of aggressive OC (Cai *et al*., 2015). Coupling between resistance to anoikis and EMT has been observed in several cancer cell lines (Frisch *et al*., 2013). Some of the common pathways shared by EMT and anoikis resistance include the PI3K‐Akt pathway (Chiarugi and Giannoni, 2008), Hippo pathway (Cordenonsi *et al*., 2011), overexpression of Notch3 (Brown *et al*., 2015), and loss of E‐cadherin (Derksen *et al*., 2006).

In this study, we use OC as the model system to explore how the *FZD7* pathway contributes to the aggressiveness of cancer cells. We found that *FZD7* expression was crucial to the maintenance of Mes phenotype, anchorage‐independent growth, and tumorigenesis. We further identified *TWIST1* as the downstream effector of *FZD7*. Manipulating *TWIST1* expression mimicked the functional consequences observed in the *FZD7* model, while *TWIST1* overexpression partially rescued the functional phenotypes abolished by *FZD7* knockdown. We subsequently identified the regulation of *TWIST1* was by *FZD7* through epigenetic modifications of H3K4me3 and H3K27ac at the *TWIST1* proximal promoter. In addition, *BCL2* expression positively correlated with *TWIST1* expression which could be from direct transcriptional regulation. Clinically, the enrichment of *FZD7*‐*TWIST1* axis correlated with poorer survival. We also provided evidence that this axis was amenable to therapeutic targeting by a small molecule porcupine (PORCN) inhibitor, C59.

## 2 Materials and methods

### 2.1 Cell culture

Ovarian cancer cell lines OVCA429 and CH1 were grown in Dulbecco's modified Eagle's medium (DMEM) supplemented with 10% FBS; OV7 and OV17R were grown in DMEM/F12 plus 10% FBS.

### 2.2 Generation of stable *TWIST1* overexpression and knockdown cell lines

For *TWIST1* overexpression, lentiviral plasmids encoding full‐length wide‐type *TWIST1* with a pLenti‐GIII‐CMV‐GFP‐2A‐Puro backbone (Applied Biological Materials Inc., Vancouver, BC, Canada) were used. For *TWIST1* knockdown, two shRNA clones (#TRCN0000020541 and #TRCN0000020542; Sigma‐Aldrich; subsidiary of Merck KGaA: St. Louis, MO, USA) were selected with pLKO.1‐puro Luciferase shRNA plasmid (#SHC007; Sigma‐Aldrich) as a control. Plasmids were mixed with MISSION® Lentiviral Packaging Mix (#SHP001; Sigma‐Aldrich) before added to a mixture of transfection reagent Fugene 6 (#11814443001; Roche, Basel, Switzerland) and OptiMEM. After 10–15 min’ incubation at room temperature, they were added to 293T cells seeded in the 6‐cm dishes. For infection, virus‐containing supernatants were harvested 48 and 72 h after transfection, filtered, and added to selected cells, together with polybrene (Sigma‐Aldrich). Twenty‐four hours after infection, cells were treated with puromycin at a proper concentration decided by their respective puromycin kill curve.

### 2.3 *BCL2* siRNA Knockdown and Generation of stable *TWSIT1*‐overexpressing OVCA429

*BCL2* small interfering RNA (siRNA; SMART pool ON‐TARGET plus), nontargeting control siRNA (ON‐TARGET plus control pool), and DharmaFECT 4 (# T‐2004‐02) transfection reagents were purchased from Dharmacon (Lafayette, CO, USA). CH1, OV17R, *TWIST1* short hairpin against FZD7‐1 (sh*FZD7‐*1), and OVCA429 *TWIST1* cells were seeded in 6‐cm dish (Corning, Corning City, NY, USA). *BCL2* expression was quantified after 72 h. Plasmid pCMV6‐AC‐tGFP‐TWSIT1 was generated by molecular cloning from pCMV6‐Entry‐TWIST1 (RC202920; OriGene, Rockville, MD, USA). TWSIT1‐overexpressing OVCA429 cells were established by transfection and then sorted into low, intermediate, and high GFP subgroups by florescence‐activated cell sorting (FACS). The high GFP subgroup cells were maintained by G418 (#10131027; Life Technologies, Carlsbad, CA, USA) at 250 μg·mL^−1^. For negative control, OVCA429 was transfected with pCMV6‐AC‐tGFP empty vector and sorted for GFP‐positive cells every time before the experiment. No stable EV‐OVCA429 survived after G418 selection.

### 2.4 Reverse transcription and quantitative PCR (RT–qPCR)

mRNA were extracted using an RNeasy mini kit (SAbiosciences, Qiagen, Hilden, Germany) according to manufacturer's protocol and reverse‐transcribed to cDNA using RT2 first‐strand kit (SAbiosciences, Qiagen). The cDNA were mixed with SYBR green master mix (SAbiosciences, Qiagen) and primers for quantitative PCR (qPCR) analysis. Five housekeeping genes *ACTB*,* B2M*,* GAPDH*,* HPRT1*, and *RPL13A* were used for normalization. Details of the primers are listed in Tables 1 and 2. For reverse transcription (RT)–qPCR data analysis, the mRNA expression of each gene was normalized to the average of the five housekeeping genes and presented as average fold change 2^−∆∆Ct^ with respect to their control. All qPCR experiments were done using ABI 7900HT (Life Technologies). Cycling conditions were set as 95 °C incubation for 10 min at the first stage, 40 cycles of a two‐step stage 95 °C for 15 s and 60 °C for 1 min, followed by dissociation stage.

**Table 1:** Details for commercially available primers (SAbiosciences, Qiagen)

Target	Cat. No.	Target	Cat. No.
*ACTB*	PPH00073E	SNAI2	PPH02475A
*B2M*	PPH01094E	TWIST1	PPH02132A
*GAPDH*	PPH00150E	ZEB1	PPH01922A
*HPRT1*	PPH01018C	ZEB2	PPH09021A
*RPL13A*	PPH01020B	CD44	PPH00114A
*SNAI1*	PPH02459A	BMI1	PPH577784A

**Table 2:** Details for other primers

	Forward primers	Reverse primers
*BCL2*	CTGCACCTGACGCCCTTCACC	CACATGACCCCACCGAACTCAAAGA
*PTEN*	CCAGTGGCACTGTTGTTTCACA	CAGGTAACGGCTGAGGGAGCTC
*BCLXL*	GATCCCCATGGCAGCAGTAAAGCAAG	CCCCATCCCGGAAGAGTTCATTCACT
*BAX*	TGG AGCTGCAGAGGATGATTG	GAAGTTGCCGTCAGAAAACATG
*ALDH1*	CTGCTGGCGACAATGGAGT	GTCAGCCCAACCTGCACAG
*CD133*	CAACCCTGAACTGAGGCAGC	TTGATAGCCCTGTTGGACCAG

### 2.5 Western blot analysis

Cell lysates were harvested using cold RIPA buffer (#R0278; Sigma‐Aldrich) with protease inhibitor (#539134) and phosphatase (#524625) inhibitor cocktails from Calbiochem, Millipore (Burlington, MA, USA). BCA assay (#23225; Thermo Scientific, Waltham, MA, USA) was performed for protein quantification. After heating, lysates were resolved by standard reducing SDS/PAGE, transferred to poly(vinylidene difluoride) membranes, blocked with 5% BSA for at least 1 h, and incubated overnight at 4 °C with the following antibodies diluted in 1% BSA in TBST: anti‐E‐cadherin (#610182) from BD Biosciences, Franklin Lakes, NJ, USA at 1 : 1000; anti‐TWIST1(#sc‐81417) from Santa Cruz Biotechnology, Dallas, TX, USA at 1 : 200; antivimentin (#M7020) from Agilent Technologies (Dako, Santa Clara, CA, USA) at 1 : 1000; anti‐actin (#A1978) and anti‐GAPDH (#G9545) from Sigma at 1 : 1000. After washing, membranes were then incubated in dark at room temperature for half an hour with Infrared dye‐conjugated secondary antibodies from Li‐COR Biosciences, Lincoln, NE, USA at 1 : 5000 diluted in TBST: IRDye 800CW goat anti‐mouse or anti‐rabbit (#926‐32210, #926‐32211) and IRDye 680LT goat anti‐mouse or anti‐rabbit (#926‐68020, #926‐68021). Blots were scanned using the Odyssey Infrared Imaging System (Li‐COR). Images were transferred to grayscale.

### 2.6 Immunofluorescence staining

Cells were seeded on 15‐mm glass coverslips (Paul Marienfeld GmbH & Co. KG, Lauda‐Königshofen, Germany) to proper confluent, fixed with 4% paraformaldehyde for 10 min, and permeabilized with 0.05% Triton X for 5 min. The fixed cells were incubated with blocking buffer (3% BSA in PBS) at room temperature for 1 h, with primary antibodies (diluted in 1% BSA in PBS) overnight at 37 °C, and then incubated with secondary antibodies for 1 h at room temperature in the dark. The primary antibodies used include anti‐E‐cadherin (#610182) from BD Transduction Laboratories; antivimentin (#M7020) from Dako; and anti‐β‐Catenin (#8480) from Cell Signaling Technology (Danvers, MA, USA). Secondary antibodies are Alexa Fluor 488‐conjugated anti‐mouse (#A11029) and Alexa Fluor 594‐conjugated anti‐mouse (#A11032) from Invitrogen (Carlsbad, CA, USA). For F‐actin staining, rhodamine phalloidin (#R415; Life Technologies) was used directly after blocking. The stained coverslips were mounted onto glass slides using Vectashield mounting medium with DAPI (#H‐1200) from Vector Laboratories (Burlingame, CA, USA). Images were taken using Nikon (Minato, Tokyo, Japan) A1R confocal microscope. To quantify immunofluorescence (IF) expression of E‐cadherin and vimentin, mean fluorescence intensity was measured by using the automated measurement function in the nikon software.

### 2.7 Spheroid formation assay

In spheroid formation assay, cells were seeded to ultralow attachment (ULA), flat‐bottom 96‐well plate (#7007; Corning) at 500 cells (OV17R and OVCA429 clones) or 200 cells (CH1 and OV7 clones) in 100 μL growth medium per well. After incubated at 37 °C, 5% CO_2_ for 10 or 14 days, calcein‐AM (#C3100MP; Life Technologies) was used to stain the living cells and ethidium homodimer‐1 (EthD‐1; #E1169; Life Technologies) was used to stain the dead cells. Images were taken and analyzed using Zeiss (Oberkochen, Germany) Axio Imager M2 imaging system. Each experiment was performed by seeding 10 wells per clone. Three independent replicate experiments were done. Only spheroids above 50 μm in diameter were included for counting. Spheroid counts were calculated by adding the total number of spheroids from 10 wells in each experiment. The data are represented by the average number of total spheroids from three experiments per 5000 (OV17R and OVCA429) and 2000 (CH1 and OV7) cells.

### 2.8 Anoikis assay (FACS and caspase 3/7 activity)

For FACS, cells were seeded in ULA 10‐cm dish (#3263; Corning) at a density of 500 000 cells per dish. After incubated at 37 °C, 5% CO_2_ for 48–72 h, cells were collected, trypsinized to get single cell suspension, and stained with propidium iodide (PI) and Annexin V (#V13242; Sigma) for 15 min at room temperature in dark. LSRII FACS analyzer was used to do the FACS with proper gating.

For the caspase 3/7 activity assay, cells were seeded in ULA 96‐well plates (#7007; Corning) at a density of 10 000 (OV17R, OVCA429, and OV7 clones) or 5000 (CH1 clones) cells per well. After 72‐h or 96‐h incubation, 20 μL of CellTiter‐Fluor reagent (for cell viability, #TB371; Promega (Madison, WI, USA)) was added to all the wells and the fluorescence was measured using a Tecan plate reader (Tecan, Männedorf, Switzerland; infinite 200) after 1‐h incubation at 37 °C. One hundred microlitee of caspase‐Glo 3/7 reagent (#TB323; Promega) was then added to all the wells, and the luminescence was measured after 1–2 h incubation at room temperature. The caspase 3/7 activities were divided by the cell viabilities and then normalized to their respective controls.

### 2.9 Molecular cloning

For *TWIST1* promoter amplification, the first pair of PCR primers were forward: GCGTATCCAAGCATTTGGAATTGGGG and reverse CTCTCGAGCGGCGACGCGTGGCCTC. The second round of PCR using primers: forward, CCGGGTACCCTTTCAAGGTCACAATGCGGAGCC and reverse, ATACTCGAGTGGGCGAGAGCTGCAGACTTGG was developed to add RE sites.

KOD hot start kit was used to do the PCR (#71086; Merck Biosciences (Novagene, Darmstadt, Germany)) with a system containing: 1× reaction buffer, 1.5 mm MgSO_4_: 0.2 mm dNTPs, 0.3 μm primers; 100 ng of Template DNA, 0.02 U·μL^−1^ KOD Hot Start DNA Polymerase and water with total reaction volume 50 μL. For PCR, step 1, polymerase activation with 95 °C for 2 min; step 2, denature with 95 °C for 20 s; step 3, annealing with lower primer *T*_m_ (°C) for 10 s; and step 4, extension with 70 °C for 30 s to 1.5 min. Totally 35 cycles of steps 2–4 were followed by a 70 °C, 4 min further extension.

After PCR, the purified PCR products and pGL3 vectors were digested with double enzymes (KpnI and XhoI; New England Biolabs, Ipswich, MA, USA) in 37 °C for 3–4 h. The digested products were then loaded into 0.8% agarose gel for gel extraction. For ligation, digested pGL3 vectors and inserts were incubated at 16 °C overnight together with T4 ligase (#M0202S, NEB) and ligation buffer. After ligation, the whole ligation system was used to do the transformation. Sequences of the inserts were validated by plasmid sequencing.

### 2.10 Promoter assay

On day 1, the cells were seeded in white 96‐well plates (#655098; Greiner Bio‐One, Kremsmünster, Austria) with 4000 cells per well and incubated at 37 °C for 24 h. On day 2, cells were transfected with pGL3‐*TWIST1*‐promoter (100 ng per well) together with pCMV‐Renilla (1.5 ng per well) and then incubated at 37 °C for 48 h. On day 4, the medium was removed from the wells and 75 μL Dual‐Glo reagent together with 75 μL fresh medium was added to each well. The plate was then put on the shaker for 30 min in dark for cell lysis, and the firefly luminescence was measured using the Tecan plate reader. After the first read, 75 μL of Dual‐Glo Stop&Glo Reagent was added to each well and incubated on the shaker in dark for another 30 min. The Renilla luminescence was then read in the same plate/well order as the firefly luminescence. For the data presentation, the value of firefly luminescence was divided by Renilla luminescence for the same well and then normalized to their respective control.

### 2.11 ChIP‐qPCR assays

Cells grown in 10‐cm dishes were cross‐linked by 1% of formaldehyde for 10 min at room temperature followed by a final concentration of 0.125 m glycine for 5 min to stop the cross‐linking. The fixed cells were washed twice with tris‐buffered saline buffer and harvested in 1 mL SDS buffer with additional protease inhibitors. The cell lysates were kept on ice for around 20 min, centrifuged at 300 ***g***, 4 °C for 10 min, reconstituted in IP buffer (0.5 v of SDS buffer and 1 v of Triton dilution buffer), and then sonicated by Bioruptor® sonifier for seven pulses (25″ on 45″ off intervals) to form DNA fragments with 200–500 bp in size. Prior to immunoprecipitation, the chromatin samples were precleared with FBS blocked protein G Sepharose beads for 1 h at 4 °C. The precleared samples were incubated with 5 μg of the following antibodies including goat immunoglobulin G (IgG) control (# sc‐2028; Santa Cruz), anti‐TWIST1 (# sc‐6070; Santa Cruz), rabbit IgG control (# sc‐2027; Santa Cruz), anti‐H3K4me3 (CS‐003‐100; Diagenode SA, Liege, Belgium), anti‐H3K27me3 (#ab6002; Abcam, Cambridge, UK), anti‐H3K27ac (#ab4729; Abcam), anti‐H3K9me3 (#ab8898; Abcam), overnight at 4 °C. The samples were then incubated with preblocked protein G Sepharose beads for 4 h at 4 °C followed by four rounds of washes. The bound DNA was eluted, de‐cross‐linked at 65 °C overnight, and subsequently purified using QIAquick PCR purification kit from Qiagen. The purified samples were subjected to qPCR analysis. For ChIP‐qPCR, the primer pairs are listed in Table 3.

**Table 3:** Details of the ChIP‐qPCR primer pairs

	Forward	Reverse
*TWIST1* promoter	CTCCTCCTCACGTCAGGCCAATG	TGGATGGCCCCGAGGTCCAAA
*BCL2* promoter region 1	GCCTCCCAAAGTGCTGAGATT	TTCGCCACCTGCTGGTTGTTT
*BCL2* promoter region 4	TTAGGACGGTGGGCCTGAAAG	CCCGAGCGTGGTGTTTACTTT
*BCL2* promoter region 5	TGTCGTAAAGCCCTTGATAAACC	TGTCGTAAAGCCCTTGATAAACC

### 2.12 Statistical analyses

For all the qPCR experiments, unpaired *t*‐test was used to analyze the expression of the certain genes in overexpression or knockdown clones compared to controls. For ChIP‐qPCR experiments, unpaired *t*‐tests were used to determine statistical significance in the enrichment of TWIST1 and the epigenetic markers at the selected DNA regions. All of the *t*‐tests were performed using graphpad prism® version 6 (GraphPad Software, La Jolla, CA, USA).

To derive the *FZD7*‐*TWIST1* signature, we extracted preprocessed gene expression data from CSIOVDB. The 77 genes whose expressions are most positively correlate the average expression of *FZD7* and *TWIST1* (Spearman correlation coefficient ρ > 0.3) were selected as an *FZD7*‐*TWIST1* signature. To compute the enrichment score of the signature, a two‐sample Kolmogorov–Smirnov‐based test was used. Kaplan–Meier analyses were performed using graphpad prism® version 5.04 (GraphPad Software). Patients were categorized into low and high groups corresponding to the first (lowest 25%) and last quartiles (highest 25%) of signature enrichment score, respectively. *P*‐values of survival analyses were computed using log‐rank test.

### 2.13 CAM xenograft

Fertilized chicken eggs were obtained from a local farm and labeled as embryonic day 0 (ED0). Eggs were wiped mechanically with dry paper towels and incubated at 37–38 °C in 60–62% humidity in a R‐COM MX‐50 incubator. On ED3, 2–3 mL of albumin was removed with 18‐G needle inserted at tip of the egg. Semipermeable adhesive films were applied at the marked upper side of shell to prevent spilling of shell particles onto the chorioallantoic membrane (CAM) while cutting the window. Small holes were made and a window cut of 1 cm^2^ in the shell using sterile sharp‐pointed surgical scissors. The windows were sealed with semipermeable adhesive films, and the eggs were kept back into the incubator. On ED9, CH1 [short hairpin luciferase (shLuci), sh*FZD7*‐1, sh*FZD7*‐2, and sh*FZD7*‐1 Twist1] and OV‐17R (shLuci, sh*FZD7*‐1, sh*FZD7*‐2, and sh*FZD7*‐1 Twist1) cells at 70% confluency were trypsinized, washed with 1× PBS, and resuspended in 100 μL of Matrigel (Corning, Cat#354234) per well. One million CH1 cells and 2 million OV17R cells were seeded onto the CAM by gently tapping the membrane with an autoclaved glass rod. The windows were again sealed with semipermeable adhesive films before keeping the eggs back in the incubator. On ED15, the tumors were cut out carefully from the CAM and the chick embryos were decapitated. The tumors were washed in 1× PBS in order to remove blood from the tumors. After washing, the tumors were kept and aligned to take images.

## 3 Results

### 3.1 *FZD7* knockdown cells demonstrate an epithelial trait

**Figure 1:**
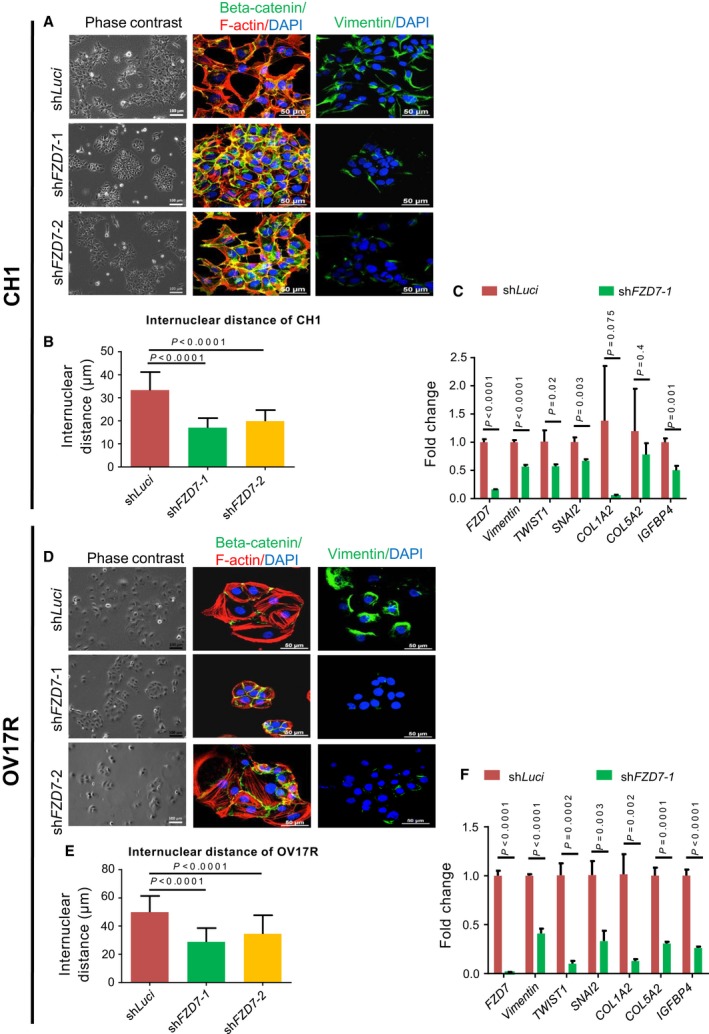
sh*FZD7* conferred an epithelial‐like phenotype and downregulation of EMT‐related genes. Morphology of (A) CH1‐shLuci, CH1‐sh*FZD7‐*1, and CH1‐sh*FZD7*‐2 clones and (D) OV17R‐shLuci, OV17R‐sh*FZD7‐*1, and OV17R‐sh*FZD7*‐2 clones shown in phase‐contrast images (left panel), IF staining (middle panel) of β‐catenin (green) and F‐actin (red), and vimentin (right panel) together with the nuclear staining of DAPI (blue). Scale bars indicated 100 μm for phase‐contrast images and 50 μm for IF images. Bar charts showing the internuclear distance (*y*‐axis, μm) of (B) CH1‐shLuci (dark red bars), CH1‐sh*FZD7*‐1 (green bars), and CH1‐sh*FZD7*‐2 (orange bars)clones (*x*‐axis) and (E) OV17R‐shLuci (dark red bars), CH1‐sh*FZD7*‐1 (green bars), and CH1‐sh*FZD7*‐2 (orange bars) clones. Error bars indicated SEM. Unpaired *t*‐tests were performed for statistical significance. Bar charts showing the fold change (*y*‐axis) of *FZD7*, vimentin, *TWIST1*,*SNAI2*,*COL1A2*,*COL5A2*, and *IGFBP4* mRNA expression (2^−∆∆Ct^) in (C) CH1‐shLuci (dark red bars), sh*FZD7*‐1(green bars) clones normalized to CH1‐shLuci and in (F) OV17R‐shLuci (dark red bars), sh*FZD7*‐1 (green bars) clones normalized to OV17R‐shLuci. mRNA expression levels were measured by qPCR normalized to a panel of housekeeping genes, *ACTB*,* B2M*,*GAPDH*,*RPL13A*, and *HPRT1*. Error bars indicated SEM. Unpaired *t*‐tests were performed for statistical significance.

Transient silencing of *FZD7* by siRNA was shown to induce colony compaction in a subset of OC cells (Asad *et al*., 2014). We confirmed this phenotypic change by using shRNA‐mediated knockdown of *FZD7* (sh*FZD7*) in two cell lines with high *FZD7* expression, CH1 and OV17R (Fig. 1C,F). sh*FZD7* cells demonstrated a more epithelial‐like morphology hallmarked by colony compaction, an increase of linear junctional staining of β‐catenin, loss of stress fibers, and a decrease in vimentin in both CH1 and OV17R cells (Fig. 1A,D). The effect of colony compaction in sh*FZD7* cells compared to their luciferase control cells was evident by the significantly decreased internuclear distance (Fig. 1B,E) in both cell lines. There was restoration of junctional staining of Pan‐cadherin (Fig. S1A) and E‐cadherin (Fig. S1B) in sh*FZD7* cells which suggested strengthening of cadherin‐based cell–cell adhesions. qPCR analysis of a panel of related EMT genes in sh*FZD7* cells showed decreases in vimentin, EMT transcription factors *TWIST1* and *SNAI2*, type I and type V collagen components *COL1A2* and *COL5A2*, and insulin‐like growth factor pathway component *IGFBP4* in both CH1 and OV17R cells (Fig. 1C,F), while significant increases in *CDH2*,* CDH3* in CH1 (Fig. S1C) and *CDH1*,* CDH3* and *EPCAM* in OV‐17R cells were observed (Fig. 1D). The demonstration of colony compaction involving cytoskeleton rearrangement, strengthening of cadherin‐based cell–cell adhesion with increased cell polarity in sh*FZD7* cells, suggested that *FZD7* mediated the maintenance of the Mes trait.

### 3.2 *FZD7* is essential for anoikis resistance and tumor formation

**Figure 2:**
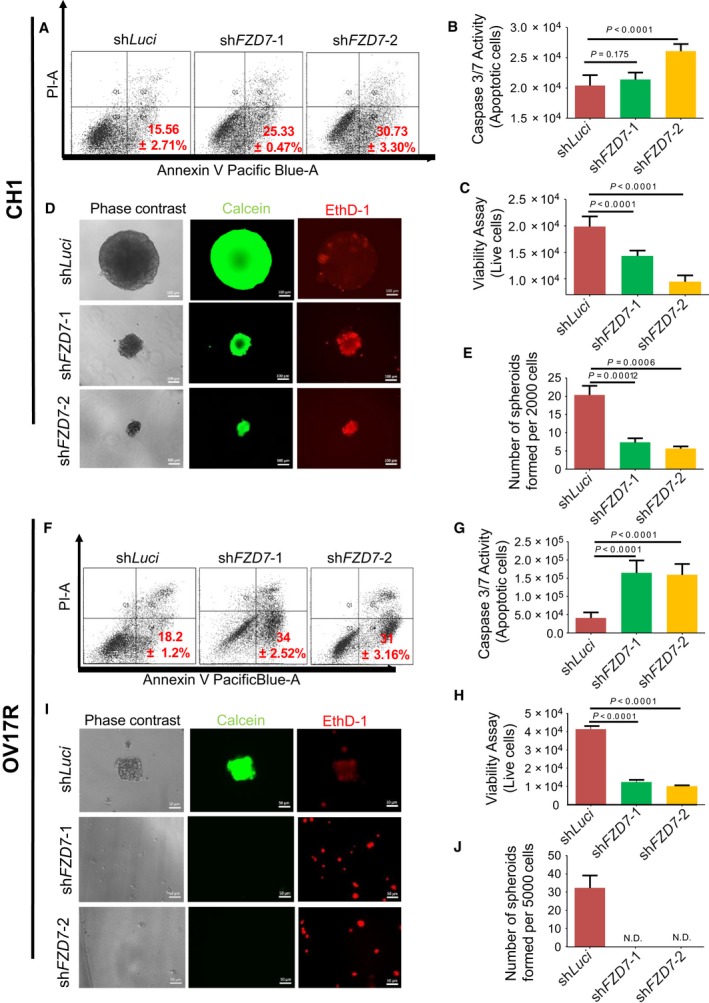
sh*FZD7* reduced the anoikis resistance and spheroid formation ability of CH1 and OV17R. Percentage of apoptotic (A) CH1‐shLuci, CH1‐sh*FZD7*‐1, and CH1‐sh*FZD7*‐2 and (F) OV17R‐shLuci, OV17R‐sh*FZD7*‐1, and OV17R‐sh*FZD7*‐2 cells after 48 h in suspension, as analyzed by flow cytometry (10 000 events). The percentage of Annexin V^+^/PI^−^ were considered apoptotic. Values (mean ± SD) in red denoted the percentage of apoptotic cells from three independent experiments. *X*‐axis: the levels of Annexin V tagged with pacific blue; *y*‐axis: the levels of PI. Bar charts showing the caspase 3/7 activities—apoptotic cells (*y*‐axis) for (B) CH1‐shLuci (dark red bars), CH1‐sh*FZD7*‐1 (green bars), and CH1‐sh*FZD7*‐2 (orange bars) cells (*x*‐axis) and (G) OV17R‐shLuci (dark red bars), OV17R‐sh*FZD7*‐1 (green bars), and OV17R‐sh*FZD7*‐2 (orange bars) cells (*x*‐axis). Viability assay showing live cells (*y*‐axis) for (C) CH1‐shLuci (dark red bars), CH1‐sh*FZD7*‐1(green bars), and CH1‐sh*FZD7*‐2 (orange bars) cells (*x*‐axis) and (H) OV17R‐shLuci (dark red bars), OV17R‐sh*FZD7*‐1 (green bars), and OV17R‐sh*FZD7*‐2 (orange bars) cells (*x*‐axis). Cells were seeded at a density of 10 000 cells per well in flat‐bottom ULA 96‐well plates for three wells per clone. After 72 h, cell viability was measured by fluorescence reading (400_Ex_/505_Em_), and cell death was measured by luminescence readout. (D) CH1‐shLuci, CH1‐sh*FZD7*‐1, and CH1‐sh*FZD7*‐2 cells were seeded at a density of 200 cells per well and (I) OV17R‐shLuci, OV17R‐sh*FZD7*‐1, and OV17R‐sh*FZD7*‐2 cells were seeded at a density of 500 cells per well in flat‐bottom ULA 96‐well plates for 10 wells per clone. After 14 days in culture, phase‐contrast images (left panel), calcein‐AM staining (middle panel) for viable cells, and EthD‐1 staining (right panel) for dead cells were analyzed. Scale bars represented 100 μm (CH1) and 50 μm (OV17R). Bar charts showing numbers of spheroids formed (*y*‐axis) in (E) CH1 clones (*x*‐axis) and (J) OV17R clones after 7 days in suspension. Only spheroids with a diameter greater than 50 μm were counted. Error bars indicated SEM. Unpaired *t*‐tests were performed for statistical significance.

Anoikis resistance, the ability to evade cell death in an environment devoid of adhesion to the ECM, is a prerequisite step for cancer metastasis, particularly during the initial phase of trans‐coelomic spread in OC (Gilmore, 2005). Following the acquisition of anoikis resistance, the ability to form spheroids or multicellular aggregates is crucial for the intraperitoneal implantation in OC. We then evaluated anoikis resistance and spheroid‐forming ability of CH1 and OV17R upon *FZD7* knockdown. *FZD7* knockdown rendered CH1 and OV17R cells more prone to anoikis, evidenced by the significant increase of Annexin V‐positive early apoptotic cells from 15.56% in CH1‐shLuci to 25.33% in CH1‐sh*FZD7*‐1 and 30.73% in CH1‐sh*FZD7*‐2 and from 18.2% in OV17R‐shLuci to 34% in OV17R‐sh*FZD7*‐1 and 31% in OV17R‐sh*FZD7*‐2 (Fig. 2A,F, respectively). We subsequently interrogated these cells for caspase 3/7 activity indicating late apoptotic events and demonstrated an increase in apoptotic activity as evidenced by an increase in caspase 3/7 activity in both CH1 and OV17R sh*FZD7* clones (except for the CH1 sh*FZD7*‐1 clone) compared to their respective luciferase controls (Fig. 2B,G). Results from the cell viability readouts also show both CH1 and OV17R sh*FZD7* clones had significantly lower numbers of live cells compared to their shLuci controls (Fig. 2C,H). When cells were seeded in an ULA 96‐well plate at a density of 200 cells per well (Materials and methods), CH1‐shLuci formed much larger spheroids with less EthD‐1‐positive death signal as compared with sh*FZD7* clones, CH1‐sh*FZD7*‐1 and CH1‐sh*FZD7*‐2 (Fig. 2D). The total number of spheroids (with diameter > 50 μm) formed significantly decreased upon *FZD7* knockdown in both cell lines (Fig. 2E,J) with OV17R‐shLuci showed the tendency to form smaller spheroids while the sh*FZD7* knockdown OV17R clones failed to form any spheroids and displayed higher EthD‐1‐positive signals (Fig. 2I). The tumorigenic efficiency was also affected by the loss of *FZD7*. By using the CAM xenograft assay (Materials and methods), tumor volume of CH1‐sh*FZD7*‐1 and CH1‐sh*FZD7*‐2 was significantly reduced (Fig. S1E) compared to CH1‐shLuci controls. Our results suggest *FZD7* is critical in cell survival and resistance to cell death in an anchorage‐independent environment which is crucial for tumorigenesis.

### 3.3 *TWIST1* confers similar functional phenotypes as *FZD7* in EMT and anoikis resistance

We next explored the possible downstream effectors of *FZD7* related to the Mes trait and anoikis resistance. Upon *FZD7* knockdown, the EMT transcription factor *TWIST1* was repressed in both CH1 and OV17R (Fig. 1C,F). *TWIST1* has been shown to be responsible for maintaining the Mes phenotype and cell survival in suspension in several cancer types (Qin *et al*., 2012). Thus, we hypothesized that *TWIST1* might be a downstream effector of *FZD7*. We first explored whether depletion of *TWIST1* resulted in a similar functional phenotype as depletion of *FZD7* in OC. Upon screening a group of relevant OC cell lines, we identified two OC cell lines OVCA429 and OV7 with appreciable low and high *TWIST1* protein expression, respectively (data not shown). OVCA429 was transfected with a GFP‐tagged *TWIST1* construct for overexpression. Three subpopulations, negative GFP, intermediate GFP, and high GFP, were subsequently sorted according to different GFP levels. These subgroups displayed a spectrum of phenotypic EMT traits with increasing levels of *TWIST1* (Fig. 2A). IF stains confirmed the levels of *TWIST1* correlated with the morphology as well as the expression of the epithelial marker, E‐cadherin, and the Mes marker, vimentin (Fig. S2B). The high GFP subgroup (*TWIST1*‐tGFP) in OVCA429 cells displayed actin reorganization with the formation of stress fibers and loss of cell–cell junction with the cytoplasmic relocalization of β‐catenin (Fig. 2B). Conversely, in OV7 cells with high *TWIST1* protein expression, loss‐of‐function experiments with knockdown of *TWIST1* using two different shRNA sequences, sh*TWIST1*‐1 and sh*TWIST1*‐2, demonstrated colony compaction compared to the dispersed morphology observed in the shLuci control of OV7 cells (Fig. 2C). IF staining of OV7 knockdown clones also demonstrated a slight restoration of E‐cadherin and a decrease in vimentin staining, which is further quantified by measuring the mean fluorescence intensity of E‐cadherin and vimentin (Fig. S2D,E). Concentrated junctional β‐catenin and epithelial‐like cortical actin bundles (Fig. 2C) were also found. The decrease of internuclear distance in OV7 sh*TWIST1*‐1 and OV7 sh*TWIST1*‐2 further indicated the increase of apical polarity (Fig. 2F).

**Figure 3:**
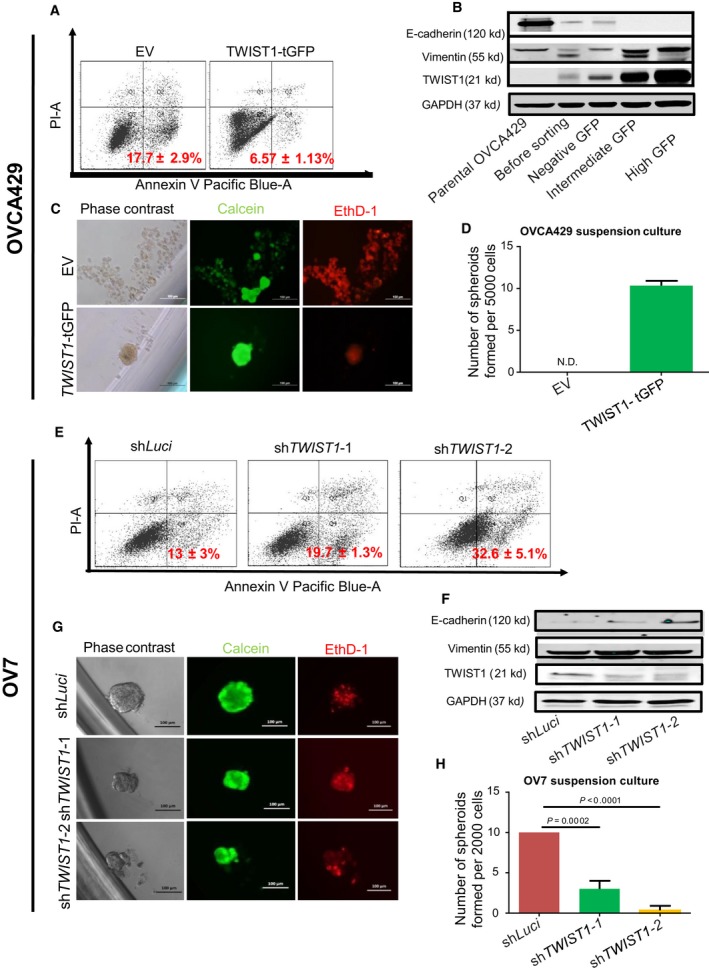
*TWIST1* played a crucial role in anoikis resistance in OVCA429 and OV7. Percentage of apoptotic (A) EV‐OVCA429 and *TWIST1*‐tGFP‐OVCA429 and (E) OV7‐shLuci, OV7‐sh*TWIST1*‐1, and OV7‐sh*TWIST1*‐2 (G) after 48 h in suspension, as analyzed by FACS (10 000 events). The percentage of Annexin V^+^/PI ^−^ were considered apoptotic. Values (mean ± SD) in red denote the percentage of apoptotic cells from three independent experiments. *X*‐axis: the levels of Annexin V tagged with pacific blue; *y*‐axis: the levels of PI. (B) Western blots for E‐cadherin, vimentin, *TWIST1*, and GAPDH in parental OVCA429, *TWIST1*‐tGFP transfected OVCA429 before FACS sorting, as well as negative GFP, intermediate GFP, and high GFP subgroup OVCA429 after sorting. (C) EV‐ and *TWIST1*‐tGFP‐OVCA429 cells were seeded at a density of 500 cells per well and (G) OV7 shLuci, sh*TWIST1*‐1, and sh*TWIST1*‐2 cells were seeded at a density of 200 cells per well in flat‐bottom ULA 96‐well plates for 10 wells per clone. After 14 days in culture, phase‐contrast images, calcein‐AM staining for viable cells, and EthD‐1 staining for dead cells were analyzed. Scale bars represented 100 μm. Bar charts showing numbers of spheroids formed by (D) OVCA429 clones *TWIST1* tGFP (green bars) or by (H) OV7 clones shLuci (dark red bars), sh*TWIST1*‐1 (green bars) and sh*TWIST1*‐2 (orange bars) after 14 days in suspension. Only spheroids with a diameter more than 50 μm were counted. Error bars indicated SEM. Unpaired *t*‐tests were performed for statistical significance. (F) Western blots for E‐cadherin, vimentin, TWIST1, and GAPDH in OV7 shLuci, sh*TWIST1*‐1, and sh*TWIST1*‐2 clones.

After 48 h in suspension, *TWIST1* overexpressed OVCA429 cells (*TWIST1*‐tGFP) had approximately 10% less apoptotic activity when compared to controls (EV‐OVCA429; Fig. 3A). Adopting the same criteria to look for spheroids with diameters more than 50 μm (Materials and methods), *TWIST1*‐tGFP‐OVCA429 cells showed spheroid formation while the control EV‐OVCA429 failed to form sizable spheroids but rather numerous smaller aggregates in suspension after 14 days (Fig. 3C,D). The data indicate that *TWIST1* overexpression increases spheroid‐forming ability of OC cells. Conversely, we investigated whether the loss of *TWIST1* expression would result in an opposite phenotype. We observed that the apoptotic population in suspension cultures increased with the knockdown of *TWIST1* in OV7 cells to 19.7% in sh*TWIST1*‐1 and 32.6% in sh*TWIST1*‐2 (Fig. 3E) compared to 13% in shLuci control cells. We also demonstrated that OV7 sh*TWIST1*‐1 and sh*TWIST1*‐2 clones formed smaller and fewer spheroids compared to the shLuci OV7 cells in three independent experiments (Fig. 3G,H). OV7 sh*TWIST1*‐2, which had better *TWIST1* knockdown efficiency, showed the least spheroid‐forming ability (Fig. 3G,H). The TWIST1 and E‐cadherin protein levels in these clones were subsequently validated using western blots (Fig. 3B,F). Collectively, these findings indicate that *TWIST1* contributes to the Mes phenotype and anoikis resistance similar to *FZD7*.

### 3.4 *TWIST1* is required for *FZD7*‐driven mesenchymal trait and anoikis resistance

**Figure 4:**
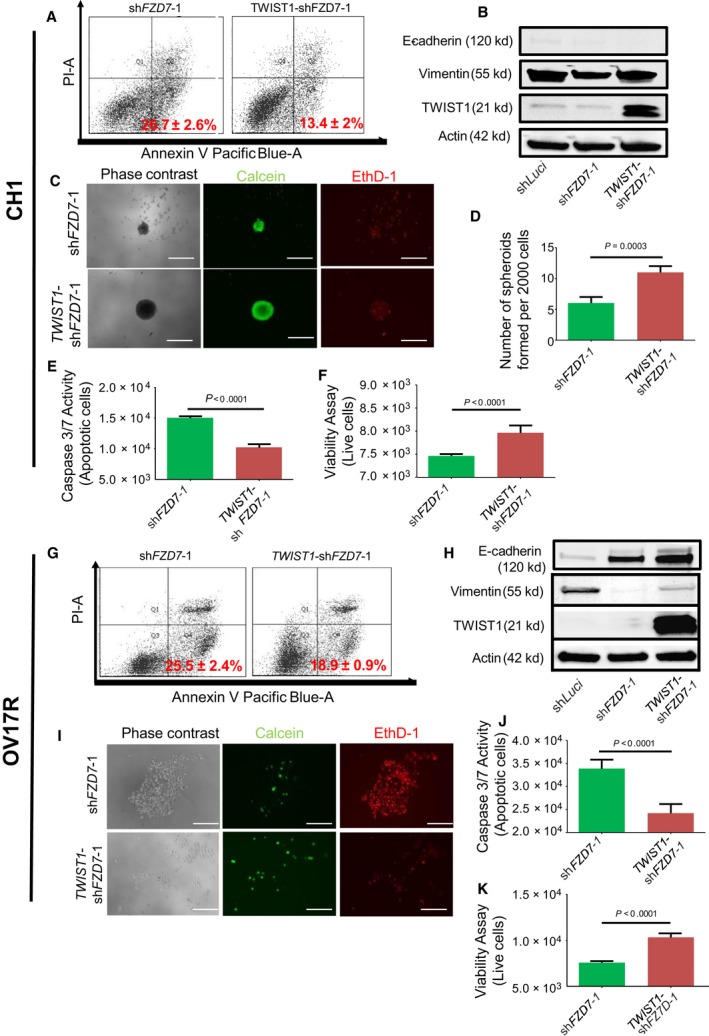
*TWIST1* rescued the anoikis resistance in sh*FZD7* clones. Percentage of apoptotic (A) CH1‐sh*FZD7*‐1 and *TWIST1*‐CH1‐sh*FZD7*‐1 and (G) OV17R‐sh*FZD7*‐1 and *TWIST1*‐OV17R‐sh*FZD7*‐1 after 72 h in suspension, as analyzed by FACS (10 000 events). The percentage of Annexin V^+^/PI ^−^ were considered apoptotic. Values (mean ± SD) in red denote the percentage of apoptotic cells from three independent experiments. *X*‐axis: the levels of Annexin V tagged with pacific blue; *y*‐axis: the levels of PI. Western blots for E‐cadherin, vimentin, TWIST1, and Actin in (B) CH1‐sh*FZD7*‐1 and *TWIST1*‐CH1‐sh*FZD7*‐1 clones and in (H) OV17R‐sh*FZD7*‐1 and *TWIST1*‐OV17R‐sh*FZD7*‐1 clones. (C) CH1‐sh*FZD7*‐1, *TWIST1*‐CH1‐sh*FZD7*‐1 cells were seeded at a density of 200 cells per well and (I) OV17R‐sh*FZD7*‐1, *TWIST1*‐OV17R‐sh*FZD7*‐1 cells were seeded at a density of 500 cells per well in flat‐bottom ULA 96‐well plates for 10 wells per clone. After 10 days, phase‐contrast images, calcein‐AM staining for viable cells, and EthD‐1 staining for dead cells were analyzed. Spheroid quantification (D) shows number of spheroids formed per 2000 cells. Scale bars represented 100 μm. Bar charts showing the caspase 3/7 activities—apoptotic cells (*y*‐axis) for (E) CH1‐sh*FZD7*‐1 (green bars), *TWIST1*‐CH1‐sh*FZD7*‐1 (dark red bars; *x*‐axis) and (J) OV17R‐sh*FZD7*‐1 (green bars), *TWIST1*‐OV17R‐sh*FZD7*‐1 (dark red bars; *x*‐axis). Viability assay showing live cells (*y*‐axis) for (F) CH1‐sh*FZD7*‐1 (green bars), *TWIST1*‐CH1‐sh*FZD7*‐1 (dark red bars; *x*‐axis) and (K) OV17R‐sh*FZD7*‐1 (green bars), *TWIST1*‐OV17R‐sh*FZD7*‐1 (dark red bars; *x*‐axis). Error bars indicated SEM. Unpaired *t*‐tests were performed for statistical significance.

To determine whether *TWIST1* is a downstream regulator of *FZD7*,* TWIST1* was overexpressed in two *FZD7* knockdown clones, CH1‐sh*FZD7*‐1 and OV17R‐sh*FZD7*‐1, to investigate whether exogenous restoration of *TWIST1* expression could reverse the effects of *FZD7* knockdown. Morphologically, *TWIST1* overexpression in CH1‐sh*FZD7*‐1 resulted in reduced colony compaction, a decrease in cell–cell junctional β‐catenin, and an increase in vimentin staining (Fig. S3A). Similarly, *TWIST1* overexpressed OV17R‐sh*FZD7*‐1 cells also showed similar changes, albeit with more marked depletion of E‐cadherin staining (Fig. 3C). In OV17R‐sh*FZD7*‐1 *TWIST1* overexpressing cells, the expression of E‐cadherin and vimentin was further quantified by measuring the mean fluorescence intensity (Fig. 3D,E). Increases in the internuclear distance were also observed in both *TWIST1* overexpressing CH1‐sh*FZD7*‐1 and OV17R‐sh*FZD7*‐1 clones (Fig. 3B,F). Meanwhile, in CH1‐sh*FZD7*‐1 cells, *TWIST1* overexpression rescued the spheroid‐forming ability of *FZD7*‐depleted CH1 cells (Fig. 4C,D). However, *TWIST1* overexpression did not appear to rescue the spheroid‐forming abilities in OV17R‐sh*FZD7*‐1 cells. Notably, we did observe an increase in calcein‐AM‐positive live cells and a decrease in EthD‐1‐positive dead cells in *TWIST1*‐OV17R‐sh*FZD7*‐1 cells (Fig. 4I). Concordant with the spheroid‐forming nature of *TWIST1* overexpressed cells, the population of Annexin V‐positive apoptotic cells decreased by 13% in CH1‐sh*FZD7*‐1 and 7% in OV17R‐sh*FZD7*‐1 upon *TWIST1* overexpression (Fig. 4A,G). In addition, there was significant reduction in caspase activity with concomitant increase of live cell signals in the *TWIST1* overexpressing CH1‐sh*FZD7* (Fig. 4E,F) and OV17R‐sh*FZD7* (Fig. 4J,K) clones. The protein expression of *TWIST1*, vimentin, and E‐cadherin was further validated using western blots (Fig. 4B,H). The data suggest that *TWIST1* rescued the anoikis resistance abolished by *FZD7* knockdown and lead to higher cell survival rates in long‐term suspension cultures.

### 3.5 *TWIST1* is regulated by epigenetic modifications downstream to *FZD7*

**Figure 5:**
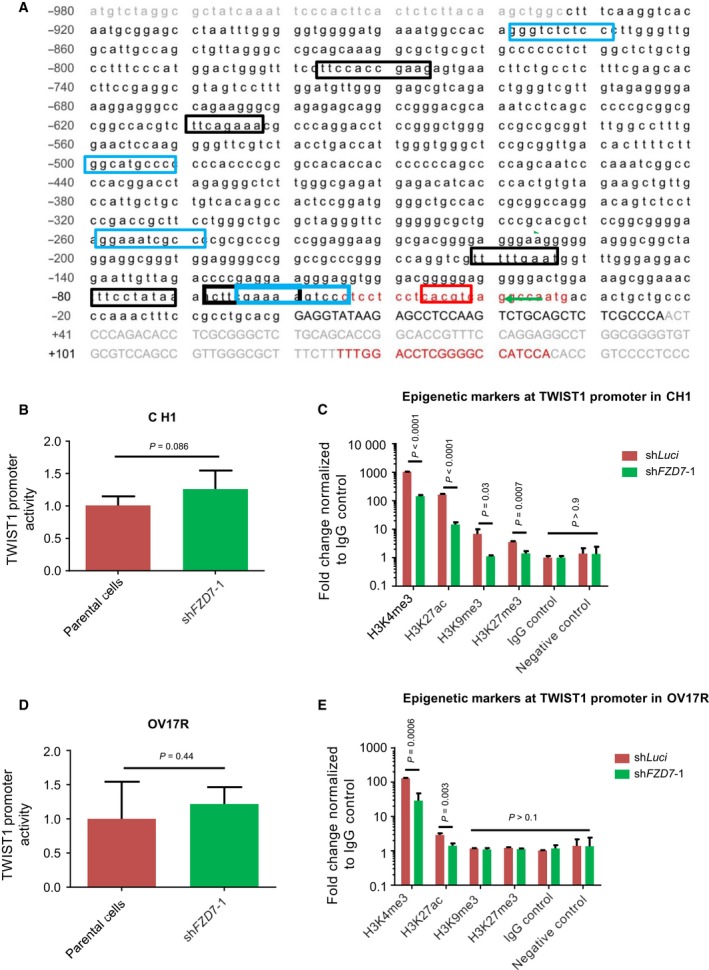
sh*FZD7* induced epigenetic modifications of *TWIST1* promoter. (A) The sequence of the *TWIST1* promoter region (black text) from −933 to +37 relative to the TSS with NF‐κB binding sites (blue box), STAT3 binding sites (black box), and an HIF1α binding site (red box). The primer sequences for histone marker ChIP‐qPCR (red text) amplifying the region from −65 to +136 were also shown. Bar charts showing the *TWIST1* promoter activities in (B) CH1‐sh*FZD7*‐1 normalized to CH1 and in (D) Ov17R‐sh*FDZ7*‐1 normalized to OV17R. Bar charts showing the enrichments represented by the fold change of percentage of input normalized to IgG control (*y*‐axis) of H3K4me3, H3K27ac, H3K9me3, H3K27ac, and IgG control (*x*‐axis) at the *TWIST1* promoter in (C) CH1‐shLuci (dark red bars) and CH1‐sh*FZD7*‐1 (green bars) and in (E) OV17R‐shLuci (dark red bars) and OV17R‐sh*FZD7*‐1 (green bars). Unpaired *t*‐tests were performed for statistical significance.

The complex regulation of *TWIST1* transcription has been reported to be regulated by stat3 (Cheng *et al*., 2008; Lo *et al*., 2007), HIF1α (Yang *et al*., 2008), and NF‐κB (Sosic *et al*., 2003) through direct binding at the *TWIST1* promoter (Fig. 5A). As such, a dual luciferase reporter assay for *TWIST1* promoter activity [−933 bp to +37 bp from transition start site (TSS)] was performed (Fig. 5A) on CH1 and OV17R parental and sh*FZD7* clones. We did not observe any significant difference in the *TWIST1* promoter activity in both CH1 and OV17R cells (Fig. 5B,D) which suggests that the suppression of *TWIST1* upon *FZD7* knockdown was not through direct repression of *TWIST1* promoter activity. As *TWIST1* regulation has previously been demonstrated to be mediated by DNA methylation within exon 1 active histone mark H3K4me3 and inactive histone mark H3K9me3 (Sakamoto *et al*., 2015), we explored whether epigenetic modifications might be an alternative mechanism of *TWIST1* regulation by *FZD7*. Previous ChIP‐sequencing data in OC (Chung *et al*., 2016) have suggested that histone modification marks were enriched at the *TWIST1* exon 1 but not at the distal promoter region. Thus, several histone marks (H3K4me3, H3K27ac, H3K9me3, and H3K27me3) at the *TWIST1* proximal promoter and exon 1 regions were interrogated via ChIP‐qPCR. A set of qPCR primers were designed for 8 regions to cover the full length of *TWIST1* exon 1. The highest enrichment of the relevant histone marks listed above was observed at the proximal promoter region of *TWIST1* (−65 bp to +136 bp from TSS) after normalizing to IgG control (Fig. 5C,E). At this region, significant enrichment of H3K4me3 and H3K27ac was observed in both CH1 and OV17R shLuci cells compared to their respective knockdown clones (sh*FZD7‐1*; Fig. 5C,E). Other histone marks, including H3K9me3 and H3K27me3, demonstrated low‐level enrichment in CH1 shLuci cells but not in OV17R shLuci cells (similar level to the IgG control) throughout the screened regions in the sh*FZD7* clones (Fig. 5C,E). Collectively, the data would suggest that *TWIST1* transcription is epigenetically controlled by active histone marks H3K4me3 and H3K27ac in CH1 and H3K4me3 in OV17R. The depletion of these marks with *FZD7* knockdown could be the mechanism which dynamically controls *TWIST1* expression.

### 3.6 *BCL2* acts as a downstream effector of the *FZD7*‐*TWIST1* axis

**Figure 6:**
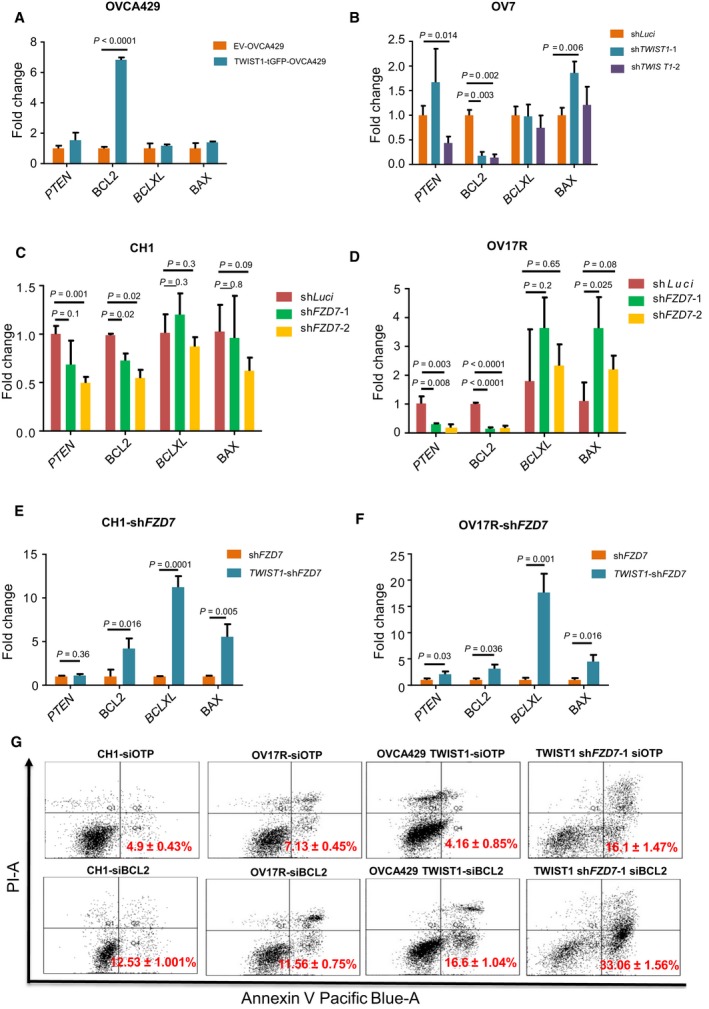
*FZD7*‐*TWIST1* regulates anoikis resistance by activating its downstream effector *BCL2*. Bar charts showing the fold change (*y*‐axis) of *PTEN*,*BCL2*,*BCLXL*, and *BAX* (*x*‐axis) mRNA expression (2^−∆Ct^) in (A) *TWIST1*‐tGFP‐OVCA429 (dark aqua bars) normalized with EV‐OVCA429 (orange bars); (B) OV7‐sh*TWIST1*‐1 (dark aqua bars) and sh*TWIST1*‐2 (purple bars) normalized with OV7‐shLuci (orange bars); (C) CH1‐sh*FZD7*‐1 (green bars), CH1 sh*FZD7*‐2 (orange bars) normalized with CH1shLuci (dark red bars); (D) OV17R‐sh*FZD7*‐1 (green bars), OV17R‐sh*FZD7*‐2 (orange bars) normalized with OV17R‐shLuci (dark red bars); (E) *TWIST1*‐CH1‐sh*FZD7* (aqua dark bars) normalized with CH1‐sh*FZD7* (dark orange bars); (F) *TWIST1*‐OV17R‐sh*FZD7* (aqua dark bars) normalized with OV17R‐sh*FZD7* (dark orange bars). mRNA expression levels are measured by qPCR normalized with a panel of housekeeping genes, *ACTB*,* B2M*,*GAPDH*,*RPL13A*, and *HPRT1*. (G) Percentage of apoptotic cells in CH1 (siOTP and si*BCL2*), OV17R (siOTP and si*BCL2*), OVCA429 TWIST1 (siOTP and si*BCL2*) and *TWIST1*‐CH1‐sh*FZD7* (siOTP and si*BCL2*) after 72 h in suspension, as analyzed by FACS (10 000 events). Values (mean ± SD) in red denoted the percentage of apoptotic cells from three independent experiments. *X*‐axis: the levels of Annexin V tagged with pacific blue; *y*‐axis: the levels of PI. Error bars indicated SEM. Unpaired *t*‐tests were performed for statistical significance.

*TWIST1* expression has been shown to inversely correlate with the tumor suppressor *PTEN* (Yin *et al*., 2010), the intrinsic apoptosis‐related pro‐apoptotic gene *BAX* (Kwok *et al*., 2005), and positively correlate with the anti‐apoptotic gene *BCL2* (Banerjee *et al*., 2012). In addition, *BCLXL* has been shown to contribute to EMT‐related resistance to apoptosis and chemotherapy (Keitel *et al*., 2014). Therefore, we screened for these genes to explore their involvement in the *FZD7*‐*TWIST1* axis. As expected, *BCL2* was upregulated in *TWIST1* overexpressed (*TWIST1*‐tGFP) OVCA429 cells and downregulated in the *TWIST1* knockdown clones of OV7 cells (sh*TWIST1*; Fig. 6A,B). When tested in other relevant cell lines, we found that *BCL2* was downregulated upon *FZD7* knockdown in CH1 and OV17R cells (Fig. 6C,D), and when *TWIST1* was overexpressed in these *FZD7* knockdown clones, CH1‐ and OV17R‐sh*FZD7*‐1, we observed not only an increase in the expression of *BCL2* but also *BCL‐XL* and *BAX* (Fig. 6E,F). To confirm whether *BCL2* regulates anoikis resistance as a downstream target of *FZD7*‐*TWIST1* axis, siRNA‐mediated knockdown of *BCL2* was performed in parental CH1 and OV17R cells, *TWIST1* overexpressing OVCA429 and CH1 sh*FZD7*‐1 cells (Fig. S4C). Anoikis assays showed that compared to the negative control siRNA pool siOTP, si*BCL2* induced significantly the Annexin V^+^/PI^−^ population (Fig. 6G). The data indicate highly correlated regulations among *FZD7*,* TWIST1*, and *BCL2* expressions. While *TWIST1* has been shown to suppress *BAX* in prostate cancer cell lines (Kwok *et al*., 2005), further work is warranted to explore whether the upregulation of *BAX* and *BCL‐XL* in *TWIST1* overexpressed CH1‐ and OV17R‐sh*FZD7* clones is indeed directly induced by *TWIST1*.

### 3.7 *FZD7*‐*TWIST1* axis correlates with poor clinical outcomes

**Figure 7:**
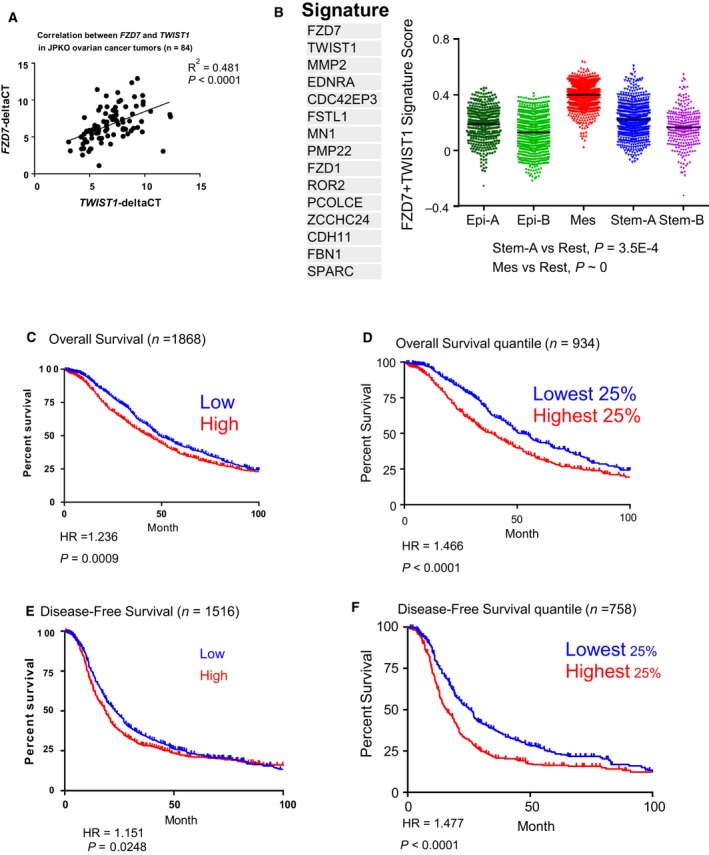
*FZD7*‐*TWIST1* signature predicts poor survival. (A) Scatter plot showing the correlation between *FZD7* (*y*‐axis) and *TWIST1* (*x*‐axis) mRNA expression in a Japanese OC cohort (GSE30311). (B) Distribution of *FZD7*‐*TWIST1* signature score (*y*‐axis) among the Epi‐A (dark green), Epi‐B (light green), Mes (red), Stem‐A (blue), and Stem‐B (purple) subtypes in CSIOVDB. Kaplan–Meier curves of median overall survival (C), disease‐free survival (E), and the highest and lowest quartiles of the *FZD7*‐*TWIST1* signature score for overall survival (D), disease‐free survivals (F) in OC patients. Stratified by the first and last quartiles of the *FZD7*‐*TWIST1* signature score.

Next, to explore the clinical relevance of the *FZD7*‐*TWIST1* axis, gene expressions for both *FZD7* and *TWIST1* were examined in several OC cohorts. Firstly, in a Japanese OC cohort (GSE30311), *FZD7* and *TWIST1* expressions were observed to positively correlate (Fig. 7A, *P* < 0.0001). Secondly, from CSIOVDB, an in‐house OC microarray database comprising of 3431 OC samples (Tan *et al*., 2015), we derived a *FZD7*‐*TWIST1* signature consisting of 15 genes with positive Spearman correlation (ρ > +0.3) to the average expression of *FZD7* and *TWIST1*. The *FZD7*‐*TWIST1* signature was significantly enriched in the Mes subtype (Fig. 7B, Kolmogorov–Smirnov test, Mes vs rest *P *=* *0). Significant differences in both overall and disease‐free survival were observed in patients stratified by either the median (Fig. 7C,E) or the highest and lowest quartiles of the *FZD7*‐*TWIST1* signature score (Fig. 7D,F). These data suggest that the *FZD7*‐*TWIST1* axis is clinically relevant in OC.

### 3.8 Targeting the *FZD7*‐*TWIST1* axis with a porcupine inhibitor

**Figure 8:**
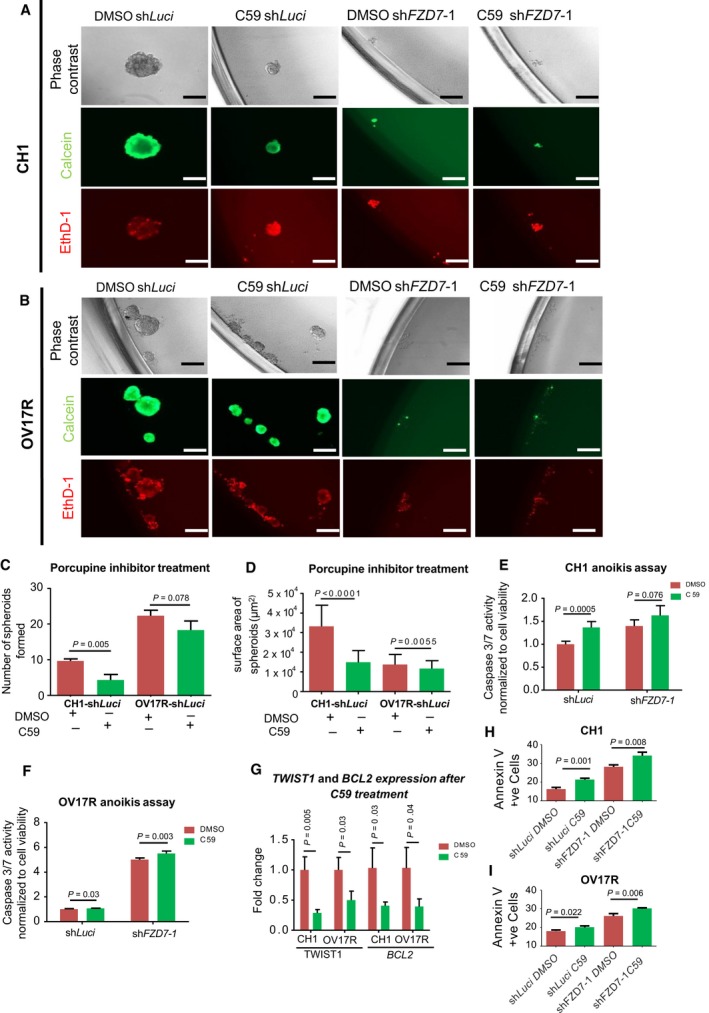
PORCN inhibitor C59 treatment in CH1 and OV17R clones. (A) CH1‐shLuci, CH1‐sh*FZD7*‐1 cells were seeded at a density of 200 cells per well and (B) OV17R‐shLuci, OV17R‐sh*FZD7*‐1 cells were seeded at a density of 500 cells per well in flat‐bottom ULA 96‐well plates for 10 wells per clone. After 10 days in culture with DMSO or C59 treatment, phase‐contrast images, calcein‐AM staining for viable cells, and EthD‐1 staining for dead cells were analyzed. Scale bars represented 200 μm. Bar charts showing (C) the numbers and (D) the surface areas of spheroids formed by CH1‐shLuci and OV17R‐shLuci with DMSO (dark red bars) or C59 (green bars) treatment for 10 days in suspension. Only spheroids with a diameter greater than 50 μm were counted. Bar charts showing caspase 3/7 activities (*y*‐axis) of DMSO (dark red bars) or C59 (green bars) treated (E) CH1‐Luci, CH1‐sh*FZD7*‐1, and (F) OV17R‐Luci, OV17R‐sh*FZD7*‐1 clones. Cells were seeded at a density of 10 000 cells per well in flat‐bottom ULA 96‐well plates for three wells per clone. After 72 h, cell viability was measured by fluorescence reading (400_Ex_/505_Em_) and cell death was measured by luminescence readout. (G) Bar charts showing the fold change of *TWIST1* (left) and *BCL2* (right) mRNA expression (2^−∆Ct^; *y*‐axis) in CH1‐shLuci and OV17R‐shLuci with DMSO (dark red bars) or C59 (green bars) treatment for 3 days. mRNA expression levels were measured by qPCR normalized with a panel of housekeeping genes, *ACTB*,* B2M*,*GAPDH*,*RPL13A*, and *HPRT1*. (H) Bar chart showing percentage of Annexin V‐positive cells in CH1‐shLuci, CH1‐sh*FZD7*‐1, (I) OV17R‐shLuci and OV17R sh*FZD7*‐1 after treating the cells with DMSO control (dark red bars) and C59 (green bars). Values (mean ± SD) for Annexin V‐positive cells denoted the percentage of apoptotic cells from three independent experiments. Error bars indicated SEM. Unpaired *t*‐tests were performed for statistical significance. C59 was used to treat cells at a final concentration of 10 nm. Unpaired *t*‐tests were performed for statistical significance.

We next explored potentially relevant therapy for targeting of the *FZD7*‐*TWIST1* axis. PORCN is an enzyme essential for palmitoylation of Wnts, and this post‐translational modification is necessary for all mammalian Wnts secretion and the binding to their receptors (Madan and Virshup, 2015). Thus, inhibition of PORCN will block the secretion and the receptor binding affinity of all human Wnts. We thus tested whether a small molecule PORCN inhibitor C59 (Proffitt *et al*., 2013) would block the functional phenotypes of the *FZD7*‐*TWIST1* axis. In CH1‐shLuci, C59 treatment resulted in a decrease in both the number and size of spheroids formed (Fig. 8A,C,D) and an increase in caspase 3/7 activity in suspension culture was observed (Fig. 8E). The level of caspase 3/7 activity was similar in C59‐treated CH1‐shLuci and DMSO‐treated CH1‐sh*FZD7*‐1 (Fig. 8E). Interestingly, C59 treatment did not further enhance caspase 3/7 activity (late apoptotic activity) in CH1‐sh*FZD7*‐1 clone (Fig. 8E). However, early apoptotic activity was observed to increase after C59 treatment as shown by an increase in Annexin V‐positive cells in the CH1 cell line (Fig. 8H). In OV17R, the effect of C59 treatment was limited both in number and in size of the spheroids formed in suspension (Fig. 8B,C,D). In line with this, C59 treatment only induced a slight increase of caspase 3/7 activity in OV17R‐shLuci and to a lesser degree as compared with untreated OV17R‐sh*FZD7*‐1 (Fig. 8F). However, C59 treatment did slightly increase caspase 3/7 activity in OV17R‐sh*FZD7*‐1 (Fig. 8F). We also observed OV17R cells showed marginal increase in Annexin V‐positive cells when treated with C59 (Fig. 8I). In both CH1 and OV17R, *TWIST1* and *BCL2* expressions were downregulated after C59 treatment (Fig. 8G); thus, the regulation of *TWIST1* and *BCL2* was, at least partially, Wnt ligand‐dependent. Of note, we observed that the alterations in the *FZD7‐TWIST1* axis do not necessarily correlate with the expression changes of the Wnt ligands. The expression of Wnt5a, a known ligand to FZD7 for the noncanonical Wnt pathway, did not show consistent patterns in either sh*FZD7* clones or in *TWIST1* rescue clones (Fig. S4A,B). Collectively, the data indicate that C59 could be utilized to mimic the functional effect of depleting *FZD7* in CH1 and to a lesser extent OV17R OC cells.

## 4 Discussion

In this study, we showed that *FZD7* and *TWIST1* expressions were associated with a Mes‐like phenotype and the expression of EMT‐related genes in OC. We also demonstrated that *FZD7* and *TWIST1* mediated resistance to cell death and formation of spheroids in suspension. The exogenous expression of *TWIST1*, at least partially, abrogated the functional effects of *FZD7* knockdown. In addition, *FZD7* regulated *TWIST1* expression via epigenetic modifications, by altering H3K4me3 and H3K27ac enrichments at the *TWIST1* promoter. These results indicate the functional importance of the *FZD7*‐*TWIST1* axis in a subgroup of OC in maintaining the Mes‐like phenotype and anoikis resistance. We further demonstrated that patients with a high *FZD7*‐*TWIST1* axis score correlated with poor survival and a small molecular weight inhibitor targeting the Wnt secretion pathway may be a potential therapeutic option.

We have previously shown that siRNA‐mediated *FZD7* transient knockdown resulted in colony compaction and a reduction in cell proliferation via casein‐kinase‐1ε mediated noncanonical Wnt/PCP pathway (Asad *et al*., 2014). Here, we show that shRNA‐mediated stable knockdown of *FZD7* not only reversed the Mes phenotype but also diminished anoikis resistance, cell survival, spheroids, and tumor formation. These results not only emphasize the importance of proper cell–matrix attachment to cell survival but also indicate crucial factors contributing to aggressiveness. *FZD7* is indeed critical for anchorage‐independent growth and tumorigenesis. We demonstrated, for the first time, the regulation of *TWIST1* by *FZD7* and the existence of the *FZD7*‐*TWIST1* axis. This proposed *FZD7*‐*TWIST1* axis is more likely to be monodirectional rather than a reciprocal feedback regulation as manipulation of *TWIST1* expression did not influence *FZD7* expression in our system (data not shown). It has been reported that *TWIST1* expression is regulated through multiple mechanisms by DNA methylation, active histone mark H3K4me3, and inactive histone mark H3K9me3 in exon 1 (Sakamoto *et al*., 2015). In OC, we found that *FZD7* regulated *TWIST1* expression mainly through affecting the active H3K4me3 mark at the *TWIST1* promoter region. This is partially in line with the study in gastric cancer cells, although no increase of H3K9me3 was observed after *FZD7* knockdown. In addition, considering that *FZD7* mediates mainly β‐catenin‐independent noncanonical Wnt signaling in EOC, the correlation among Wnt signaling, EMT, and anoikis resistance in our model further suggests the importance of targeting EMT‐related factors. *ZEB1*, a classic EMT transcription driver downstream to the β‐catenin‐dependent canonical Wnt signaling, has been shown to be essential for the tumorigenic potential of mantle cell lymphoma with anti‐apoptotic genes *MCL1* and *BCL2* acting as its downstream effectors (Sánchez‐Tilló *et al*., 2014). The role of *TWIST1* in our system is similar to what has been described in *ZEB1*. This suggests the importance of EMT transcription drivers in Wnt signaling mediated cell survival in addition to their classic roles in repressing E‐cadherin expression (Thiery *et al*., 2009).

The Wnt pathway is known to regulate apoptosis through the canonical pathway (Chen *et al*., 2001). Among the cell death‐related genes screened in this study, we observed that *BCL2* expression consistently correlated with *TWIST1* expression. *BCL2* has also been shown to correlate with activation of Wnt pathway in several studies (Fani *et al*., 2016; Wang *et al*., 2012; Yang *et al*., 2015; Zhang *et al*., 2015). Interestingly, knockdown of *WNT5A*, a known ligand for *FZD7*, decreases the expression of *BCL2* (Zhang *et al*., 2015). There have been studies to suggest that Wnt signaling is pro‐apoptotic and activation of the canonical Wnt signaling by conditional expression of an active form of β‐catenin would downregulate *BCL2* and elevate caspase 3/9 activity leading to a loss of mitochondrial membrane potential resulting in increase in apoptosis in hematopoietic stem/progenitor cells (Ming *et al*., 2012). Thus, the correlation of Wnt signaling and apoptosis might be context dependent and this should be taken into consideration when we evaluate the role of targeting Wnt signaling.

The treatment with PORCN inhibitor, C59, revealed that CH1 was more sensitive than OV17R in functional blockade of anoikis resistance. However, in both CH1 and OV17R cells, C59 treatment significantly decreased the expression of *TWIST1* and *BCL2* (Fig. 8G). This suggests that the PORCN inhibitor could still induce downstream transcriptional changes similar to knocking down of *FZD7* in both CH1 and OV17R cells. However, OV17R may be intrinsically resistant to C59 or to PORCN inhibitors in general. If the first hypothesis is true, C59 resistance can be overcome by using an alternative best‐in‐class PORCN inhibitor. Further testing of other PORCN inhibitors in development is warranted for confirmation. DMSO‐OV17R‐sh*FZD7*‐1 cells were more prone to anoikis as compared with C59‐OV17R‐shLuci. This suggests that if the later was true, this could result from either the presence of crosstalk between the Wnt pathway with other more dominant pathways, or from the Wnt ligand‐independent role of *FZD7* in regulating anoikis resistance. The presence of crosstalk signals between *FZD7*‐*TWIST1* axis and other pathways that may contribute to anoikis resistance is intriguing and requires further investigation. For example, Hedgehog signaling, which is aberrantly activated in several types of cancers, linked Wnt signaling pathways with cell survival genes *BCL2* and EMT driver genes *SNAI1/2*,* ZEB1/2* through epigenetic or genetic alterations during carcinogenesis (Katoh and Katoh, 2009). The Wnt ligand‐independent role of *FZD7* has not been reported previously.

## 5 Conclusions

In conclusion, we demonstrate that the *FZD7*‐*TWIST1* axis is involved in the maintenance of Mes phenotype, resistance to anoikis and tumor formation through epigenetic modifications. We also identified *BCL‐2* is as a downstream effector of this axis. Furthermore, we provided *in vitro* evidence that PORCN inhibitors can be used to inhibit this axis to suppress aggressiveness of OC cells that are dependent on *FZD7*‐*TWIST1* axis.

## Author contributions

RYH, MT, MA, and VH involved in conception and design. MA, MT, MKW, and RYH involved in development of methodology.MA, MT, VH, KT, and JY involved in acquisition of data. MA, MT, VH, RYH, and TTZ involved in analysis and interpretation of data. MA, MT, VH, and RYH involved in writing, review, and/or revision of manuscript. RH, CS, and JPT involved in study supervision.

## Supporting information


**Fig. S1.** Knockdown of *FZD7* results an increase in cadherin based cell‐cell adhesion at cell junction.
**Fig. S2.** *TWIST1* overexpression shows similar phenotype change as compared to *FZD7* with an increase of Mes marker and loss of epithelial marker.
**Fig. S3.** *TWIST1* regulates epithelial Mes transition.
**Fig. S4.** *TWIST1* and Wnt5a expression in shLuc and shFZD7 cells.
