# Supplementary material for: The FZD7‐TWIST1 axis is responsible for anoikis resistance and tumorigenesis in ovarian carcinoma
Source: Mol Oncol. 2019 Jan 19;13(4):757–80. doi: 10.1002/1878-0261.12425 (PMC6441896; doi:10.1002/1878-0261.12425)

Supplementary Figure 1

a

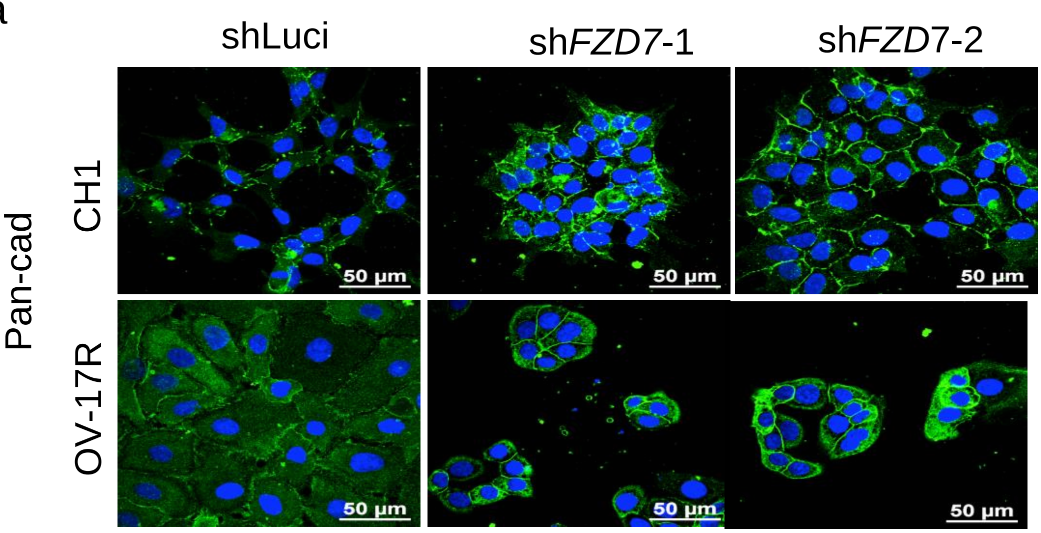

b

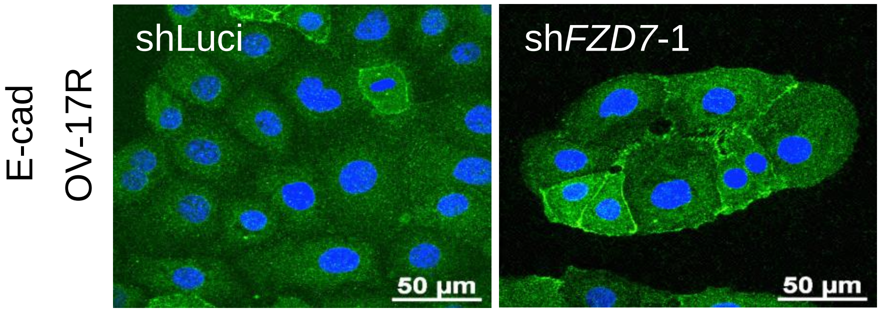

c

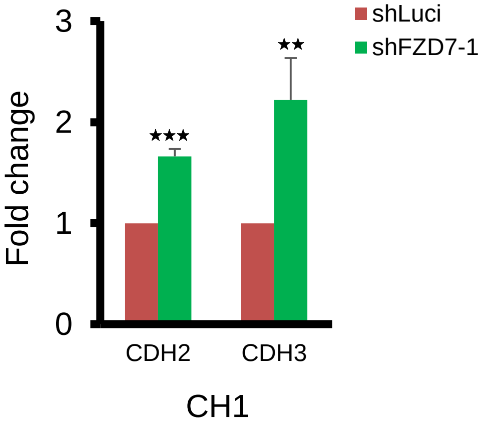

d

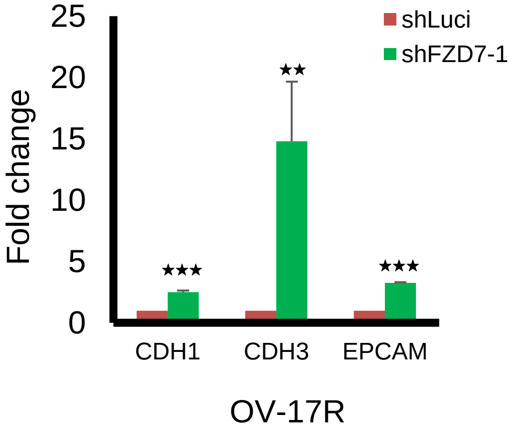

e

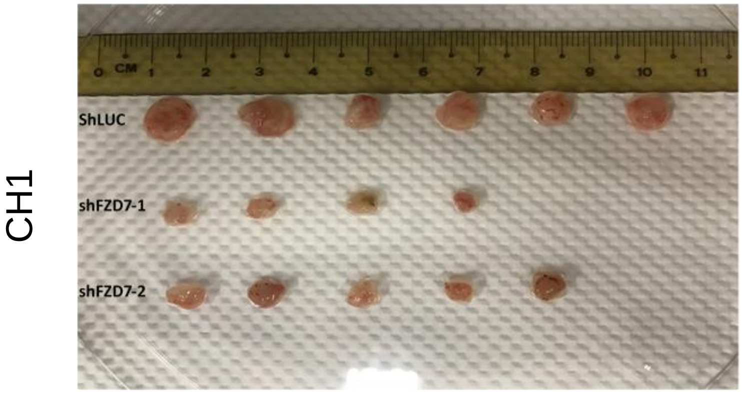

P-value * <0.05
** <0.01
***<0.001

Supplementary Figure 2

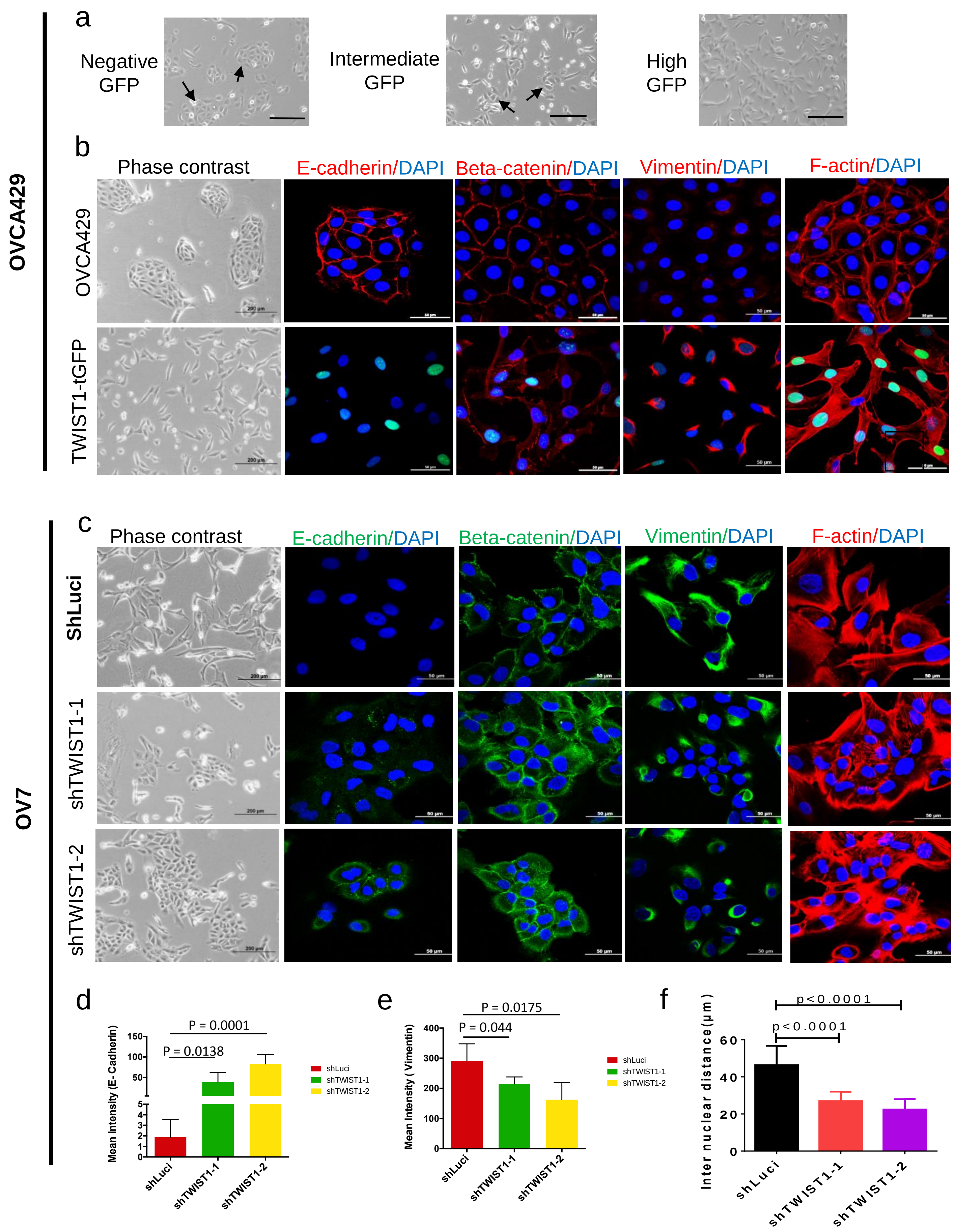

Supplementary Figure 3

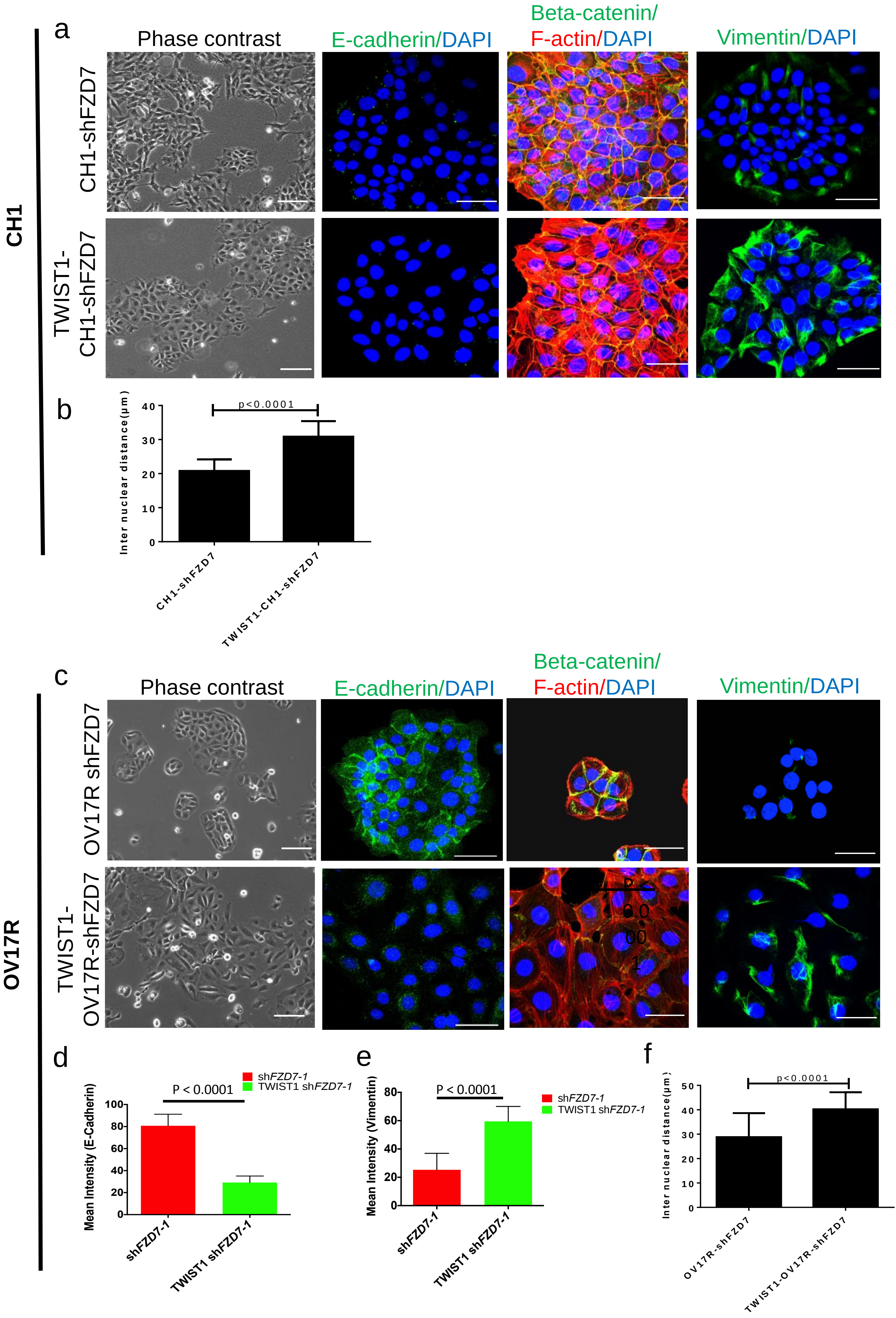

Supplementary Figure 4

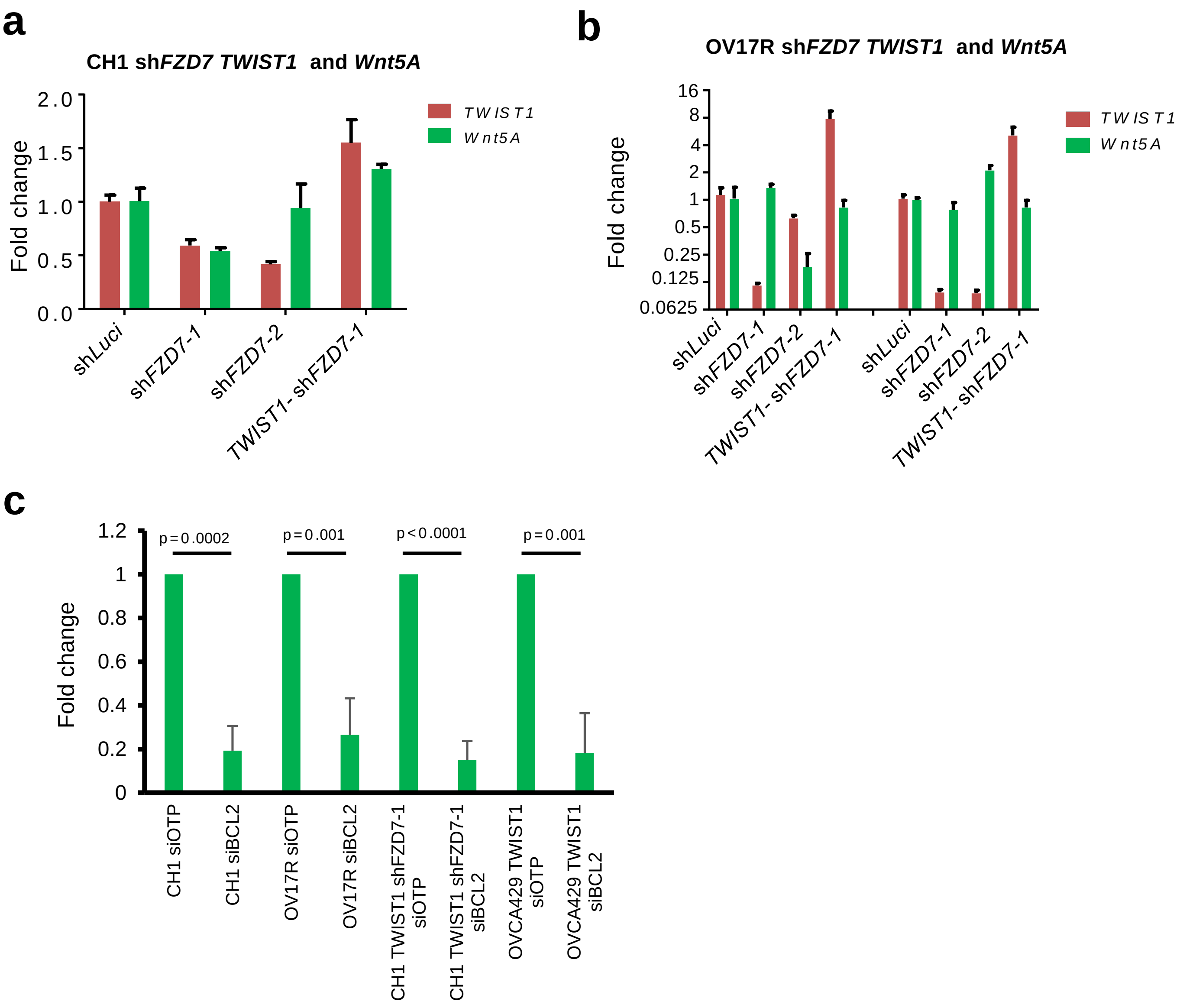

Supplement: Supplementary file 1 — Fig. S1. Knockdown of FZD7 results an increase in cadherin based cell‐cell adhesion at cell junction. Fig. S2. TWIST1 overexpression shows similar phenotype change as compared to FZD7 with an increase of Mes marker and loss of epithelial marker. Fig. S3. TWIST1 regulates epithelial Mes transition. Fig. S4. TWIST1 and Wnt5a expression in shLuc and shFZD7 cells. [file MOL2-13-757-s001.pdf]
