# Supplementary material for: The FZD7‐TWIST1 axis is responsible for anoikis resistance and tumorigenesis in ovarian carcinoma
Source: Mol Oncol. 2019 Jan 19;13(4):757–80. doi: 10.1002/1878-0261.12425 (PMC6441896; doi:10.1002/1878-0261.12425)
Supplement: Supplementary file 2 [file MOL2-13-757-s002.docx]

## **Supplementary Figure Legends:**

Fig. S1. (a) Immunofluorescence staining of Pan-cadherin in CH1 (shLuci, sh*FZD7*-1and sh*FZD7*-2), OV17R (shLuc, sh*FZD7*-1and sh*FZD7*-2) and (b) E-cadherin in OV17R shLuci and sh*FZD7*-1 cells. (c) qPCR expression of CDH2, CDH3 in CH1(shLuc and sh*FZD7*-1) and (d) CDH1, CDH3 and EPCAM in OV17R (shLuci and sh*FZD7*-1) cells, n=3 in triplicates. (e) Image of CH1 cells shLuci, sh*FZD7*-1 and sh*FZD7*-2 tumour formed inside the CAM. Scale bars represented 50 µm. Error bars indicated SEM. Unpaired T-tests were performed for statistical significance.

Fig. S2. (a) Phase contrast image of *TWIST1* overexpressed GFP tagged OVCA429 cells sorted by FACS according to their GFP expression level representing Negative GFP, Intermediate GFP and High GFP. (b) Phase contrast image of OVCA429 and *TWIST1* overexpressed OVCA429 cells (left), Immunofluorescence staining of E-cadherin, β-catenin, Vimentin, DAPI and F-actin. (c) Phase contrast image of OV7 shLuci, sh*TWIST1*-1 and sh*TWIST1*-2 (left), Immunofluorescence staining of E-cadherin, β-catenin, Vimentin, DAPI and F-actin. (d) Mean Fluoresce intensity of E-cadherin and (e) Vimentin in OV7 shLuci, sh*TWIST1*-1 and sh*TWIST1*-2 cells, n=4 mean fluorescence staining of E-cadherin and Vimentin. (f) Bar chart showing the internuclear distance of OV7 cells in shLuci, sh*TWIST1*-1 and sh*TWIST1*-2, n=200 internuclei distance. Scale bars represented for Phase contrast image are 200 µm and for Immunofluorescence is 50 µm. Error bars indicated SEM. Unpaired T-tests were performed for statistical significance

Fig. S3. (a, c) Phase contrast image (left) of sh*FZD7* cells and *TWIST1* overexpressedsh*FZD7* cells in CH1 and OV17R cells, Immunofluorescence staining of E-cadherin, F-actin, β-catenin, DAPI and Vimentin (right). (b, f) Internuclear distance of CH1 and OV17R in sh*FZD7* and *TWIST1*-sh*FZD7* cells, n=200 internuclei distance. Mean Fluoresce intensity of E-cadherin (d) and Vimentin (e) in OV17R (sh*FZD7* and *TWIST1* sh*FZD7*), n=7 mean fluorescence staining of E-cadherin and Vimentin. Scale bars represented for Phase contrast image is 200 µm and for Immunofluorescence is 50 µm. Error bars indicated SEM. Unpaired T-tests were performed for statistical significance

Fig. S4. (a, b) Fold change qPCR expression of *TWIST1* and Wnt5a in CH1 (shLuci, sh*FZD7*-1, sh*FZD7*-2 and *TWIST1*-sh*FZD7*-1) and OV17R (shLuci, sh*FZD7*-1, sh*FZD7*-2 and *TWIST1*-sh*FZD7*-1), n=3 in triplicates. (c) Bar chart showing knockdown efficiency of *BCL2* in parental CH1, PA1 and CH1 *TWIST1*-sh*FZD7*-1, OVCA429 *TWIST1* quantified by Qpcr, n=3 in triplicates. Error bars indicated SEM. Unpaired T-tests were performed for statistical significance
